# Sestrin2 remedies podocyte injury via orchestrating TSP-1/TGF-β1/Smad3 axis in diabetic kidney disease

**DOI:** 10.1038/s41419-022-05120-0

**Published:** 2022-07-30

**Authors:** Shan Song, Chonglin Shi, Yawei Bian, Zhaohua Yang, Lin Mu, Haijiang Wu, Huijun Duan, Yonghong Shi

**Affiliations:** 1 Department of Pathology, Hebei Medical University, Shijiazhuang, China; 2 Center of Metabolic Diseases and Cancer Research, Institute of Medical and Health Science, Hebei Medical University, Shijiazhuang, China; 3 Hebei Key Laboratory of Kidney Diseases, Shijiazhuang, China; 4 The Second Hospital of Hebei Medical University, Shijiazhuang, China

**Keywords:** Kidney diseases, Molecular biology

## Abstract

Sestrin2 is identified as a stress-induced protein and could functionate in many aspects. In our study, we investigated the latent impact of Sestrin2 on podocyte injury and its molecular mechanism in vivo and in vitro in diabetic kidney disease (DKD). Sestrin2 was low-expressed in renal biopsies from individuals with DKD, the glomeruli from diabetic mice, and mouse podocytes exposed to high glucose (HG). Sestrin2 overexpression ameliorated HG-induced phenotypic alterations, apoptosis, and oxidative stress in conditionally immortalized mouse podocytes and modulated the activity of Thrombospondin-1 (TSP-1)/transforming growth factor (TGF-β1)/Smad3 pathway in podocytes. Moreover, TSP-1 inhibitor LSKL or TGF-β blocker Pirfenidone arrested podocyte injury induced by HG. Streptozotocin (STZ) was employed to render equivalent diabetes in B6-TgN (CMV-Sestrin2) (TgN) and wild-type (WT) control mice. Sestrin2 alleviated increased levels of 24‐h urinary protein, blood urea nitrogen, serum creatinine and triglyceride, and urine 8-OHdG in diabetic mice. Podocyte phenotypic alterations, increased expression of apoptosis-associated proteins and podocyte loss were observed in WT but not in diabetic TgN mice, as well as oxidative stress. Additionally, TSP-1/TGF-β1/Smad3 signaling pathway was also suppressed in glomeruli of diabetic TgN mice. Thus, Sestrin2 mitigates podocyte injury in DKD via orchestrating TSP-1/TGF-β1/Smad3 pathway, underlining Sestrin2 as a promising therapeutic target for DKD.

## Introduction

Podocytes are critical constituents of the glomerular filtration apparatus; furthermore, the integrity of podocytes and their foot processes, along with the slit diaphragm, is vital for inhibiting proteinuria [1, 2]. Previously, clinical and basic research demonstrated that podocyte injury is a principal determinant of diabetic kidney disease (DKD) [3, 4]. Podocytes originate from cells undergoing mesenchymal-to-epithelial transition (MET); however, these cells can undergo epithelial-to-mesenchymal transition (EMT), a reverse form of embryogenesis characterized by loss of podocyte-specific marker expression and gain of transitional marker expression in the kidneys under pathological conditions [5]. Since renal tubular epithelial cells are likely to undergo EMT after chronic injury, we hypothesized that podocyte EMT had a strong effect on podocyte dysfunction, which might lead to glomerular filtration deficiency. In addition, previous studies have shown that high glucose (HG) can induce renal podocyte apoptosis [6–8]. Therefore, it is clinically important to identify potential therapeutic targets to efficiently ameliorate podocyte injury.

Although hyperglycemia is considered as the driving force of DKD development, some studies indicate that hyperglycemia might not be the dominant underlying culprit in DKD [9, 10]. Evidence suggests that oxidative stress is closely linked to renal dysfunction in DKD [11, 12]. The Sestrin family is a group of highly conserved proteins that have a variety of biological functions and whose expression can be induced by stress [13]. Sestrin2, which is known as hypoxia-inducible gene 95, is an important member of Sestrin family that can be cloned from A172 human glioma cell line and is located on human chromosome 1p35.3 [14]. Sestrin2 can be activated by various metabolic stresses, including hypoxia, DNA damage, oxidative stress, and endoplasmic reticulum stress [14]. Additionally, Sestrin2 plays a critical role in mitigating the accumulation of reactive oxygen species (ROS), maintaining energy balance, enhancing autophagy, reducing protein synthesis, and slowing the progression of metabolic diseases [15]. Recently, novel functions of Sestrin2 and their relevant mechanisms have been reported; notably, the role of Sestrin2 in DKD has been emphasized. Sestrin2 overexpression may relieve albumin-induced EMT in renal tubular epithelial cells [16]. In addition, HG can downregulate the expression of Sestrin2 and decrease the phosphorylation level of adenylate-activated protein kinase (AMPK), resulting in Nox4-dependent endothelial nitric oxide synthase dysfunction and glomerular mesangial cell fibrosis [17]. Thus, these studies indicate that Sestrin2 may be involved in the pathogenesis of DKD, but the mechanism is still unclear.

Here, we identified a novel role for Sestrin2 in modulating HG-induced podocyte injury via thrombospondin-1 (TSP-1)/transforming growth factor-β1 (TGF-β1)/Smad3 axis. Similar results were observed in diabetic mice. These results suggest that Sestrin2 might serve as a new therapeutic target for treating podocyte injury in DKD.

## Results

### The level of Sestrin2 is diminished in glomeruli from mice with DKD and renal biopsies from individuals with DKD

**Fig. 1:**
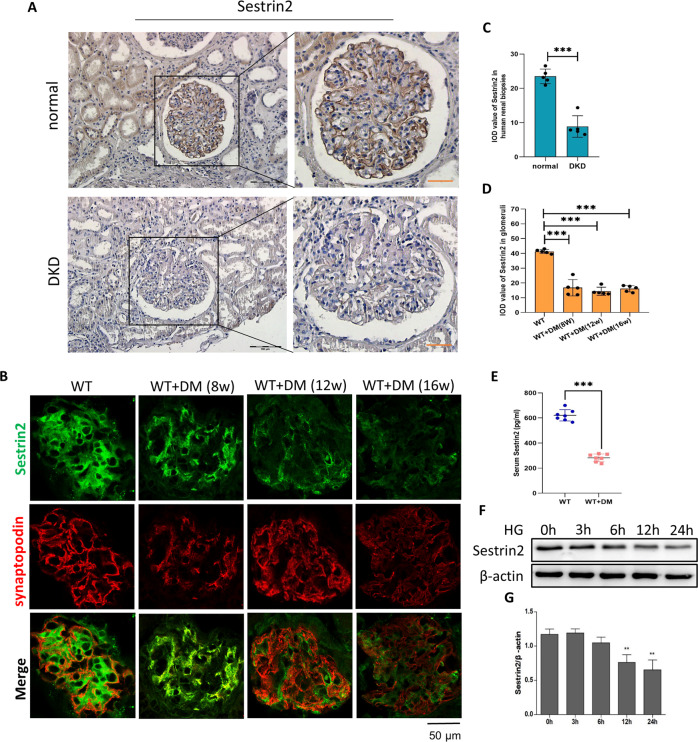
The level of Sestrin2 is decreased in glomeruli from mice with DKD and renal biopsies from individuals with DKD. **A** Representative immunohistochemical staining images of Sestrin2 in renal biopsies from normal subjects and diabetic kidney disease (DKD) patients. Scale bar: black 100 μm, red 50 μm. **B** Representative immunofluorescence images of Sestrin2 (green) and synaptopodin (red) in glomeruli from wild-type (WT) mice and STZ-induced diabetic (WT + DM) mice of 8 weeks, 12 weeks or 16 weeks. **C** Quantification of Sestrin2 in human renal biopsies (five glomeruli were analyzed, *n* = 5). **D** Quantification of Sestrin2 in glomeruli from all groups of mice (five glomeruli were analyzed, *n* = 5). **E** The levels of Sestrin2 were examined by ELISA from serum of WT mice and mice of WT + DM group (*n* = 7). **F**, **G** The protein level of Sestrin2 was detected by western blot in mouse podocytes exposed to high glucose (HG) at time courses of 0, 3, 6, 12 and 24 h (**P < 0.01 vs HG 0 h, *n* = 3). Data are expressed as mean ± SD. *P < 0.05, **P < 0.01, ***P < 0.001.

The immunohistochemical staining results showed that the expression of Sestrin2 was decreased in glomeruli and renal tubules of DKD patients compared to those of normal subjects (Fig. 1A, C). Subsequently, double immunofluorescence staining was used to assess the expression of Sestrin2 in the glomeruli of wild-type (WT) mice and streptozotocin (STZ)-induced diabetes model mice. Synaptopodin is an actin-binding protein that is expressed in crucial cell compartments, such as neuronal dendritic spines in the brain and kidney podocyte foot processes [18]. Here, synaptopodin was used to label renal podocytes in mice. Notably, Sestrin2 expression was downregulated in the glomeruli of diabetic mice at 8, 12, and 16 weeks (Fig. 1B, D). Additionally, we observed decreased serum levels of Sestrin2 in diabetic mice compared with WT mice (Fig. 1E). In vitro, Sestrin2 expression was examined in cultured mouse podocytes exposed to HG for different times (Fig. 1F, G). The protein level of Sestrin2 gradually decreased with time and tended to be the lowest at 24 h (Fig. 1F, G).

### Sestrin2 ameliorates podocyte phenotypic alterations due to HG

**Fig. 2:**
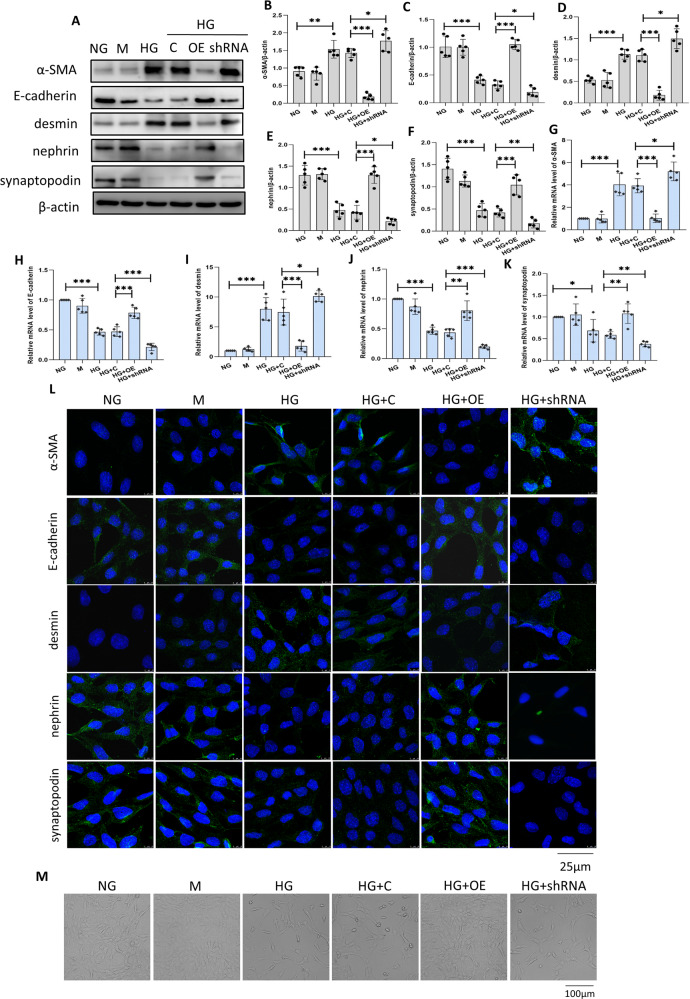
Sestrin2 attenuates podocyte phenotypic alterations induced by HG. **A**–**F** A representative Western blot and relevant quantification of α-SMA, E-cadherin, desmin, nephrin, and synaptopodin in mouse podocytes (*n* = 5). **G**–**K** The mRNA levels of α-SMA, E-cadherin, desmin, nephrin, and synaptopodin were analyzed by RT-qPCR in mouse podocytes (*n* = 5). **L** Representative immunofluorescence images of α-SMA, E-cadherin, desmin, nephrin, and synaptopodin in mouse podocytes, DAPI is a dye for nucleus. **M** Morphological changes of podocytes cultured under different conditions were analyzed by the inverted microscope. NG: 5.6 mM D-glucose; M: 5.6 mM d-glucose+24.4 mM mannitol; HG: 30 mM d-glucose; HG + C: HG + control shRNA plasmid; HG + OE: HG + Sestrin2 pcDNA; HG + shRNA: HG + Sestrin2 shRNA plasmid. Data are expressed as mean ± SD. *P < 0.05, **P < 0.01, ***P < 0.001. α-SMA α-smooth muscle actin, mRNA messenger RNA, RT-qPCR quantitative real-time polymerase chain reaction.

To ascertain whether Sestrin2 affects podocyte injury, podocytes were transfected with Sestrin2 pcDNA plasmid, Sestrin2 shRNA plasmid or control plasmid and then incubated with a high concentration of glucose for 24 h. As a result, the protein levels of α-SMA and desmin were significantly increased by HG, while E-cadherin, nephrin and synaptopodin levels were decreased by HG compared with those in the normal glucose (NG) group (Fig. 2A–F). Apparently, Sestrin2 overexpression reversed the changes in these proteins, and Sestrin2 silencing augmented HG-induced changes in the expression of these proteins (Fig. 2A–F). The PCR results were consistent with the Western blot results (Fig. 2G–K), and immunofluorescence staining revealed similar effects (Fig. 2L). Photos were taken by an inverted microscope to observe the morphological changes in podocytes from all groups (Fig. 2M). As is shown in Fig. 2M, podocytes in NG and NG + mannitol groups exhibited a spreading, arborized appearance with processes (Fig. 2M). Podocytes in HG and HG + C groups exhibited an elongated morphology (Fig. 2M). Sestrin2 overexpression improved HG-induced morphological changes in podocytes, while Sestrin2 shRNA plasmid exacerbated HG-induced morphological alterations (Fig. 2M).

### Sestrin2 mitigates podocyte apoptosis induced by HG

**Fig. 3:**
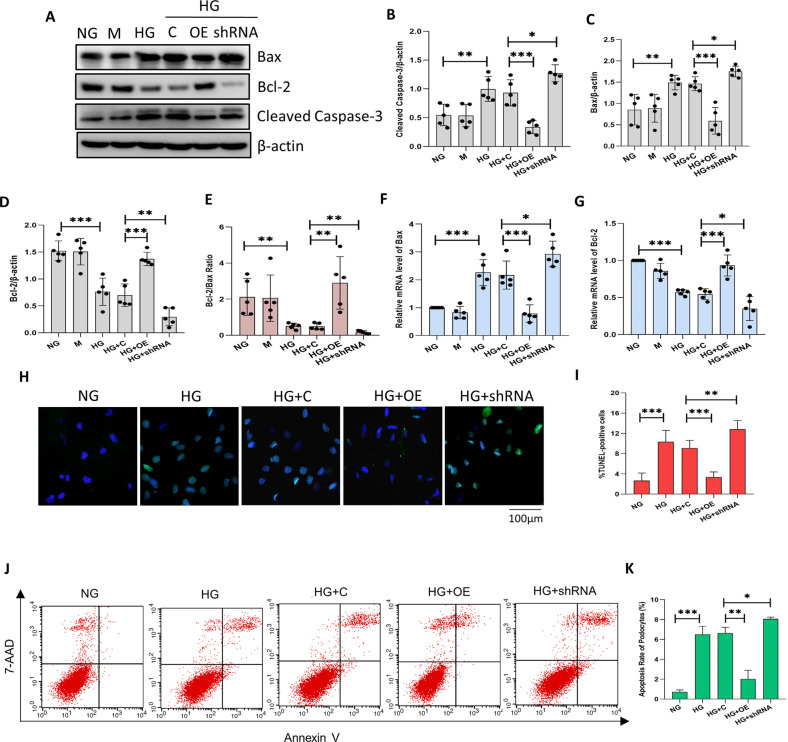
Sestrin2 alleviates podocyte apoptosis induced by HG. **A**–**D** A representative western blot and relevant quantification of Bax, Bcl-2 and Cleaved Caspase-3 in mouse podocytes (*n* = 5). **E** The relative expression of Bcl-2/Bax ratio in mouse podocytes. **F**, **G** The mRNA levels of Bax and Bcl-2 were analyzed by RT-qPCR in mouse podocytes (*n* = 5). **H**, **I** TUNEL staining results for podocytes under different conditions (*n* = 5). **J**, **K** The apoptosis rate of podocytes was detected by flow cytometry (*n* = 3). NG: 5.6 mM d-glucose; M: 5.6 mM d-glucose+24.4 mM mannitol; HG: 30 mM d-glucose; HG + C: HG + control shRNA plasmid; HG + OE: HG + Sestrin2 pcDNA; HG + shRNA: HG + Sestrin2 shRNA plasmid. Data are expressed as mean ± SD. *P < 0.05, **P < 0.01, ***P < 0.001. TUNEL terminal deoxynucleotidyl transferase-mediated dUTP nick-end labeling.

Afterward, we examined whether Sestrin2 could affect HG-induced podocyte apoptosis. Apparently, there were increases in Bax and Cleaved Caspase-3 protein levels and decreases in Bcl-2 protein levels in podocytes cultured under HG conditions compared with those in NG group (Fig. 3A–E). Sestrin2 overexpression efficiently prevented these HG-induced changes in apoptosis-associated proteins, while Sestrin2 knockdown worsened these HG-induced changes (Fig. 3A–E). Likewise, PCR results showed similar changes of Bax and Bcl-2 (Fig. 3F, G). TUNEL staining showed that there were many more apoptotic podocytes in HG group than in NG group (Fig. 3H, I). There was a reduction in the apoptotic podocytes transfected with Sestrin2 pcDNA, while silencing Sestrin2 increased the number of apoptotic podocytes (Fig. 3H, I). Furthermore, there was a higher apoptosis rate in podocytes in HG group than in NG group, and this effect was mitigated by Sestrin2 overexpression and accentuated by Sestrin2 knockdown (Fig. 3J, K).

### Sestrin2 interacts with TSP-1 in podocytes in response to HG exposure

Proteomic analysis was performed to better elucidate the underlying mechanism of the protective effect of Sestrin2 against HG-induced podocyte injury. A total of 36 distinct proteins were screened by means of mass spectrometry, including TSP-1, Collagen alpha 1 (I) chain, basement membrane-specific heparan sulfate proteoglycan core protein and so on (Fig. S1A). We used GO enrichment analysis and KEGG pathway analysis to further identify the relevant signaling pathways and found that 15 pathways were prominently enriched (Fig. S1B). Sestrin2 overexpression significantly downregulated the expression of TSP-1, which was mainly enriched in TGF-β signaling pathway; thus, TSP-1 and TGF-β signaling pathway were anchored (Fig. S1B). Furthermore, co-immunoprecipitation (Co-IP) and immunofluorescence were conducted to examine whether Sestrin2 could interact with TSP-1. The results showed that there was an interaction between Sestrin2 and TSP-1 (Fig. S1C, D).

### Sestrin2 hampers HG-induced oxidative stress and activation of TSP-1/TGF-β1/Smad3 signaling pathway in podocytes

**Fig. 4:**
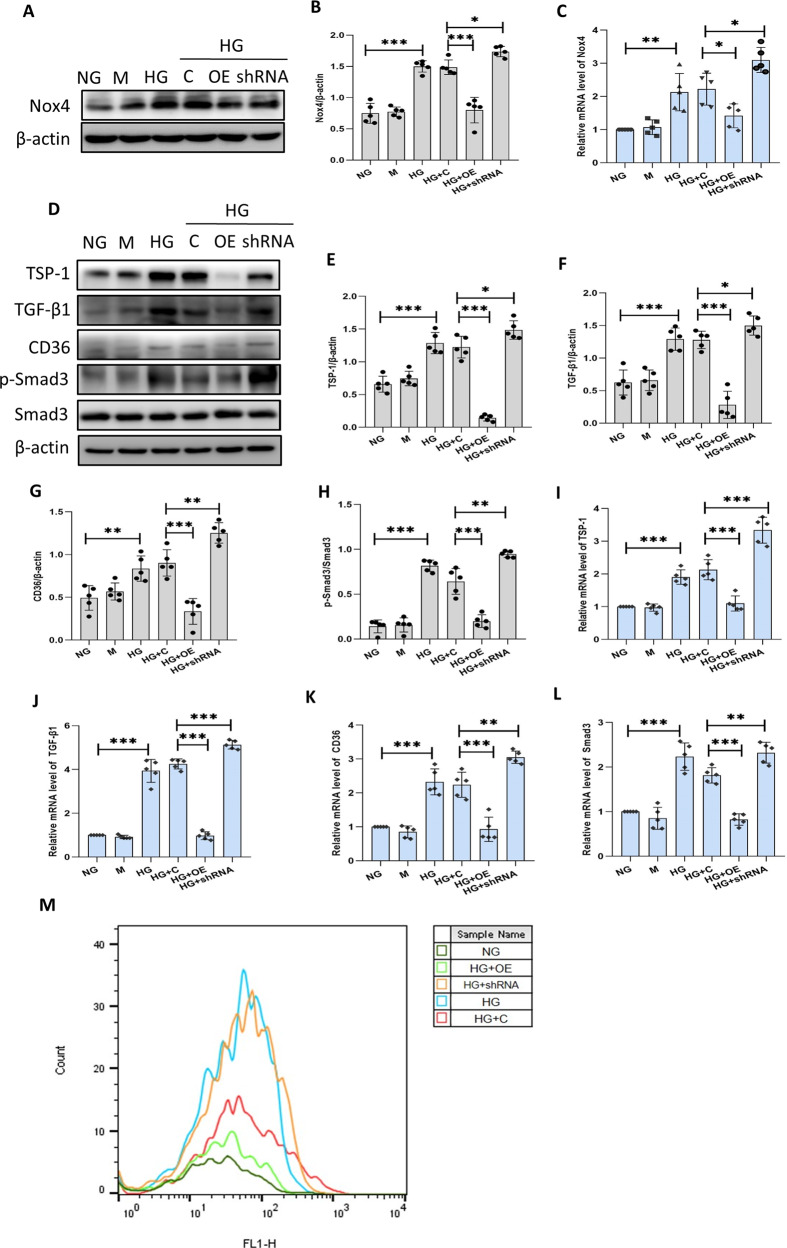
Sestrin2 ameliorates HG-induced oxidative stress and activation of TSP-1/TGF-β1/Smad3 signaling pathway in podocytes. **A**–**C** The protein and mRNA level of Nox4 was detected by western blot and RT-qPCR in podocytes (*n* = 5). **D**–**H** The protein levels of TSP-1, TGF-β1, CD36, and phosphorylation level of Smad3 were examined by western blot in podocytes (*n* = 5). **I**–**L** RT-qPCR was used to detect the mRNA levels of TSP-1, TGF-β1, CD36, and Smad3 in podocytes (*n* = 5). **M** ROS production of podocytes cultured under different conditions was determined by flow cytometry. NG: 5.6 mM d-glucose; M: 5.6 mM d-glucose+24.4 mM mannitol; HG: 30 mM d-glucose; HG + C: HG + control shRNA plasmid; HG + OE: HG + Sestrin2 pcDNA; HG + shRNA: HG + Sestrin2 shRNA plasmid. Data are expressed as mean ± SD. *P < 0.05, **P < 0.01, ***P < 0.001.

It remains unclear whether Sestrin2 affects oxidative stress and activation of TSP-1/TGF-β1/Smad3 pathway; thus, oxidative stress and TSP-1/TGF-β1/Smad3 pathway were investigated. The protein and mRNA levels of Nox4 were elevated in HG group compared to NG group, and these effects were suppressed by Sestrin2 overexpression and markedly exacerbated by Sestrin2 silencing (Fig. 4A–C). Besides, DCHF-CA was used to examine intracellular ROS levels in podocytes from different groups. ROS levels in podocytes were higher in HG group than NG group, and this effect was reduced by Sestrin2 overexpression and exacerbated by Sestrin2 silencing (Fig. 4M). In addition, we examined the protein expression of TSP-1, TGF-β1, and CD36, as well as the phosphorylation level of Smad3 (Fig. 4D–H). The levels of TSP-1, TGF-β1, CD36 and phosphorylated Smad3 were higher in podocytes of HG group than those in NG group, and the effects were inhibited by Sestrin2 overexpression and exacerbated by Sestrin2 silencing (Fig. 4D–H). Additionally, PCR results confirmed the Western blot results (Fig. 4I–L).

### NAC exerts an inhibitory effect on HG-induced podocyte EMT and apoptosis and the activation of TSP-1/TGF-β1/Smad3 pathway

Served as an antioxidant, NAC was utilized to clarify whether ROS inhibition could relieve HG-induced podocyte EMT and apoptosis. As a result, NAC restrained podocyte EMT and apoptosis, as well as the activation of TSP-1/TGF-β1/Smad3 pathway induced by HG (Figs. S2 and S3).

### Suppressing TSP-1 arrests podocyte phenotypic alterations and apoptosis aroused by HG

We utilized TSP-1 inhibitor LSKL to determine whether TSP-1 inhibition could protect against HG-induced podocyte injury. Consequently, HG-induced podocyte injury was notably inhibited by LSKL (Figs. S4 and S5).

### TGF-β inhibition alleviates podocyte EMT and apoptosis induced by HG

Pirfenidone (a blocking agent) was used to further examine whether inhibiting TGF-β/Smad3 would affect HG-induced phenotypic alterations and apoptosis in podocytes. In consequence, Pirfenidone exerted a protective effect against HG-induced podocyte injury (Figs. S6 and S7).

### Sestrin2 prevents glomerular manifestations in STZ-induced diabetic mice

**Fig. 5:**
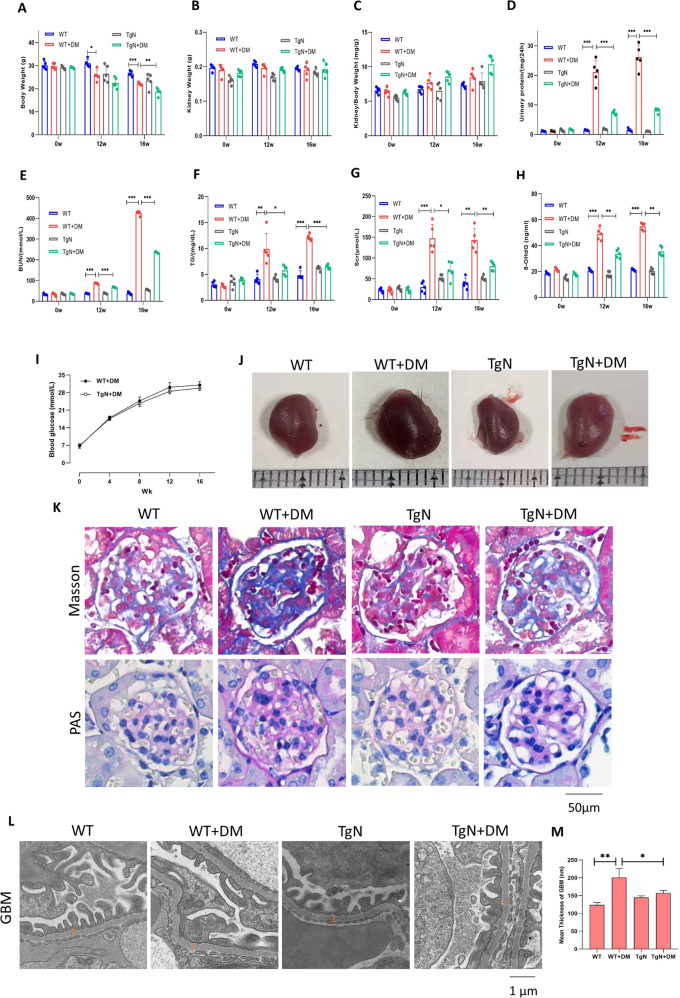
Sestrin2 prevents glomerular manifestations in STZ-induced diabetic mice. **A**–**C** The body weight, kidney weight, and kidney/body weight ratio were obtained from mice of WT, WT + DM, TgN and TgN+DM groups, which were sacrificed 12 weeks or 16 weeks after the onset of diabetes (*n* = 5). **D**–**H** The Urinary protein (24 h), BUN, TG, Scr, and 8-OHdG were detected from urine or serum of mice of four different groups, which were sacrificed 12 weeks or 16 weeks after the onset of diabetes (*n* = 5). **I** The blood glucose levels of four different groups of mice were examined every four weeks (*n* = 5). **J** The representative photographs of kidneys were captured from four groups of mice. **K** The representative Masson and PAS staining images of renal tissues of mice from four groups. **L**, **M** Transmission electron microscopy assay showed the ultrastructure of glomeruli in renal cortex from four groups of mice (*n* = 3). Orange lines referred to the thickness of the glomerular basement membrane (GBM). Magnification: ×10,000. STZ streptozotocin, WT wild-type mice, WT + DM diabetic WT mice, TgN B6-TgN (CMV-Sestrin2) mice, TgN + DM diabetic TgN mice, BUN blood urea nitrogen, TG triglyceride, Scr serum creatinine, 8-OHdG: 8-hydroxy-2 deoxyguanosine; PAS periodic acid-schiff. Data are expressed as mean ± SD. *P < 0.05, **P < 0.01, ***P < 0.001.

With the purpose of investigating the role of Sestrin2 in DKD, STZ was used to induce type 1 diabetes in WT and B6-TgN (CMV-Sestrin2) (TgN) mice. Subsequently, mice were sacrificed 12 weeks or 16 weeks after the onset of diabetes. Biochemical parameters and renal measurements, including body weight, kidney weight, kidney/body weight, Upro, BUN, TG, Scr and 8-OHdG, were measured and shown in Fig. 5A–H, and blood glucose levels were examined every four weeks during the experiment, as shown in Fig. 5I (Fig. 5A–I). The body weight of diabetic WT mice was lower than that of control WT mice at 12 weeks, but there was no obvious difference between diabetic TgN mice and diabetic WT mice (Fig. 5A). At 16 weeks, not only did diabetic WT mice have lower body weights than WT mice, but the body weights of diabetic TgN mice were lower than those of diabetic WT mice (Fig. 5A). However, there were no significant differences in kidney weight or kidney/body weight ratio (Fig. 5B, C). Regarding Upro, BUN, TG, Scr and 8-OHdG, there were prominent differences between diabetic WT group and WT group (Fig. 5D–H). These indices were much higher in diabetic WT mice than in WT mice, but lower in diabetic TgN mice (Fig. 5D–H). Interestingly, blood glucose levels in two diabetic groups showed no differences throughout the 16-week experimental period, indicating that Sestrin2 might not lower blood glucose levels (Fig. 5I).

Notably, diabetic mice showed kidney enlargement, which was ameliorated by Sestrin2 (Fig. 5J). Masson and PAS staining were performed to examine the glomerular manifestations of mice. We found that diabetic mice showed noticeable renal pathological changes compared with WT mice, including a slightly widened mesangial area, irregular thickening of the glomerular basement membrane (GBM), increased extracellular matrix (ECM) and partial glomerular capillary luminal stenosis; these effects were reversed by Sestrin2 gene modification (Fig. 5K). Besides, Sestrin2 alleviated foot process effacement and abnormal GBM thickening induced by diabetes, as shown by transmission electron microscopy (TEM) (Fig. 5L, M). These findings indicate that Sestrin2 protects kidneys from glomerular manifestations of DKD.

### Sestrin2 reverses phenotypic alterations and increased expression of apoptosis-associated proteins in podocytes and reduces the number of podocytes in diabetic mice

**Fig. 6:**
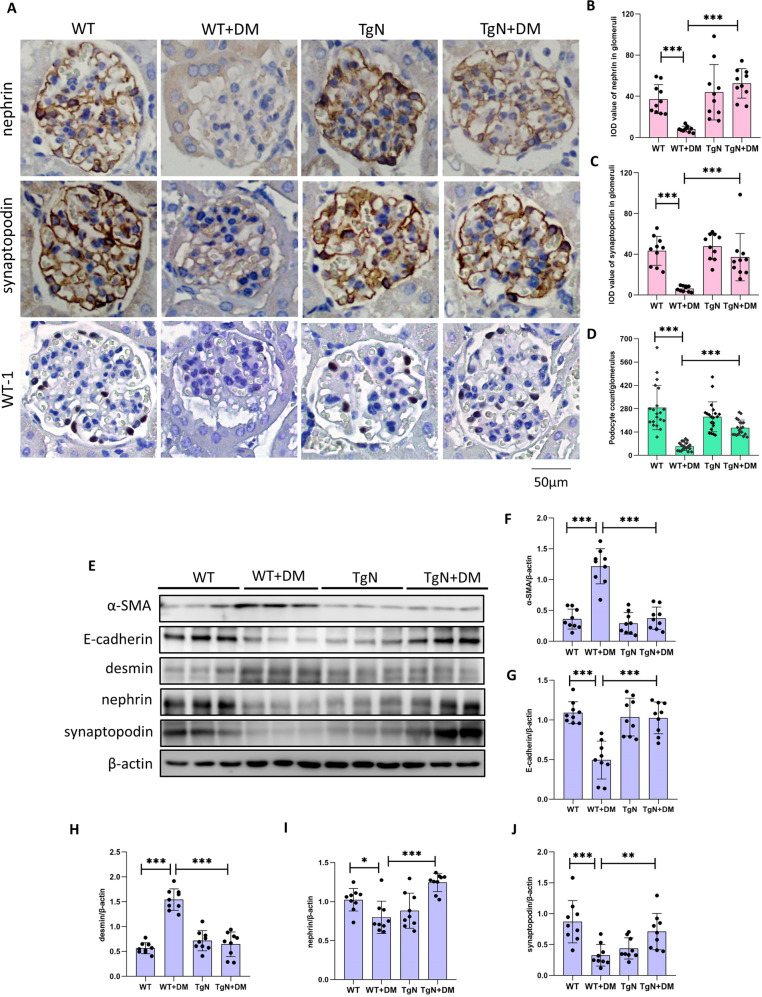
Sestrin2 reverses podocyte phenotypic alterations along with reduction in podocyte number of diabetic mice. **A**–**D** The expression of nephrin, synaptopodin and WT-1 of glomeruli from WT, WT + DM, TgN and TgN+DM mice were detected by immunohistochemistry (For nephrin and synaptopodin, 10 glomeruli were analyzed; and for WT-1, 20 glomeruli were analyzed. *n* = 5). **E**–**J** A representative Western blot and relevant quantification of α-SMA, E-cadherin, desmin, nephrin and synaptopodin in glomeruli from mice of four different groups (n = 3). WT wild-type mice, WT + DM diabetic WT mice, TgN B6-TgN (CMV-Sestrin2) mice, TgN+DM diabetic TgN mice, WT-1 Wilms’ Tumor 1. Data are expressed as mean ± SD. *P < 0.05, **P < 0.01, ***P < 0.001.

In order to elucidate the role of Sestrin2 in podocyte injury, we assessed podocyte phenotypic alterations, the expression of apoptosis-associated proteins in podocytes and the change in podocyte number in renal tissues of mice. The immunohistochemistry results showed that nephrin and synaptopodin levels were decreased in diabetic WT mice compared with nondiabetic WT mice and were increased in diabetic TgN mice (Fig. 6A–C), and the Western blot results showed similar trends (Fig. 6E, I, J). Additionally, the expression of α-SMA and desmin was increased and the protein expression of E-cadherin was decreased in glomeruli of diabetic WT mice compared with that in WT ones (Fig. 6E–H). Whereas, these alterations were reversed in diabetic TgN group (Fig. 6E–H). Likewise, double immunofluorescence staining for α-SMA and E-cadherin in glomeruli of mice revealed similar results (Fig. S8A, B). Podocyte loss might indicate the progression to advanced disease because podocytes are components of the glomerular filtration barrier [19, 20]. Therefore, we examined the change in podocyte number by staining for Wilms’ tumor antigen-1 (WT-1), which is a transcription factor restricted to podocytes [21]. We found that the podocyte number was markedly reduced in diabetic WT mice compared to WT mice and that this decrease was minimal in diabetic TgN mice (Fig. 6A, D).

**Fig. 7:**
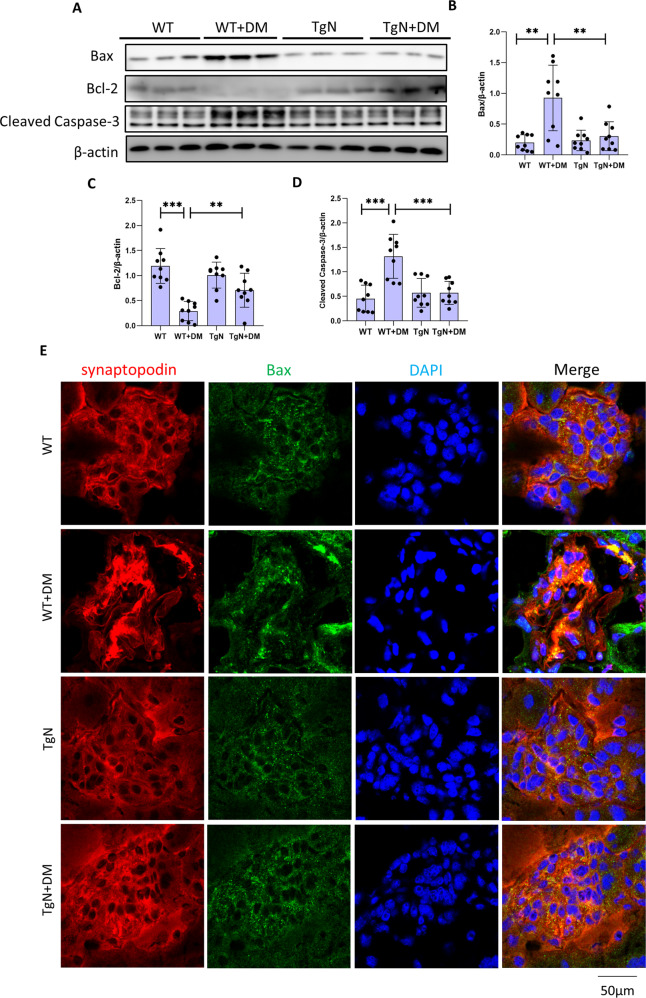
Sestrin2 reverts increased expression of apoptosis-associated proteins in podocytes of diabetic mice. **A**–**D** A representative Western blot and relevant quantification of Bax, Bcl-2, and Cleaved Caspase-3 in glomeruli from mice of WT, WT + DM, TgN and TgN+DM groups (*n* = 3). **E** Representative immunofluorescence images of Bax (green) and synaptopodin (red) in glomeruli from four groups of mice, and DAPI is a dye for nucleus. WT wild-type mice, WT + DM diabetic WT mice; TgN: B6-TgN (CMV-Sestrin2) mice; TgN + DM diabetic TgN mice. Data are expressed as mean ± SD. **P < 0.01, ***P < 0.001.

Next, we examined the expression of apoptosis-associated proteins in the glomeruli of kidney tissues from mice. The protein levels of Bax and Cleaved Caspase-3 were markedly increased and Bcl-2 protein levels were decreased in the glomeruli of diabetic WT mice compared with those of WT mice, as shown in Fig. 7A–D. Meanwhile, the change in the expression of these proteins was reversed in diabetic TgN group (Fig. 7A–D). Then, we performed double immunofluorescence staining and found that the results were consistent with the Western blot results (Fig. 7E, Fig. S9A, B).

### Sestrin2 modifies oxidative stress and activation of TSP-1/TGF-β1/Smad3 signaling pathway induced by diabetes

**Fig. 8:**
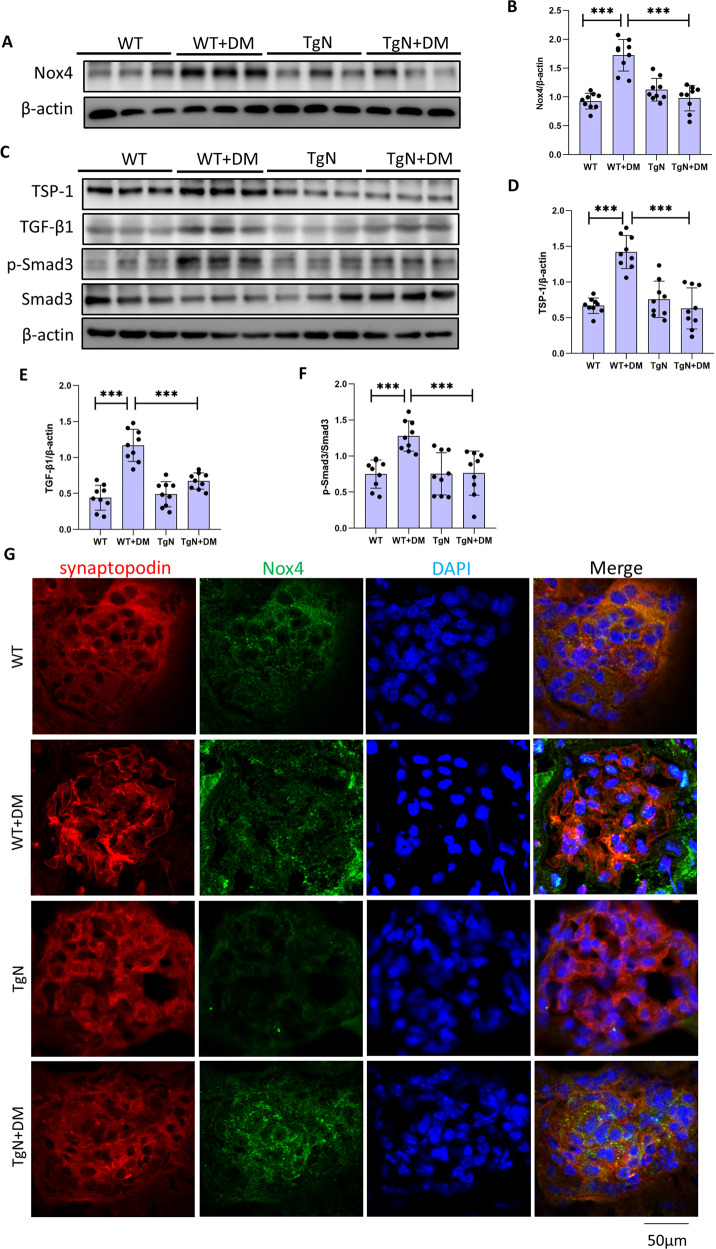
Sestrin2 modulates oxidative stress and activation of TSP-1/TGF-β1/Smad3 signaling pathway induced by diabetes. **A**–**F** A representative Western blot and relevant quantification of Nox4, TSP-1, TGF-β1, and phosphorylation level of Smad3 in glomeruli from mice of WT, WT + DM, TgN, and TgN+DM groups (*n* = 3). **G** Representative immunofluorescence images of Nox4 (green) and synaptopodin (red) in glomeruli from four groups of mice, and DAPI is a dye for nucleus. WT wild-type mice, WT + DM diabetic WT mice, TgN B6-TgN (CMV-Sestrin2) mice, TgN+DM diabetic TgN mice. Data are expressed as mean ± SD. ***P < 0.001.

We further examined the mechanism of DKD. We demonstrated that Sestrin2 suppressed the increase in 8-OHdG levels induced by diabetes in mice (Fig. 5H). Nox4 protein expression in glomeruli of diabetic WT group was much higher than that in WT group and was downregulated in diabetic TgN mice (Fig. 8A, B). In addition, the expression of Nox4 in podocytes was obviously increased in diabetic WT mice and decreased in diabetic TgN mice, suggesting that Sestrin2 ameliorates podocyte injury by reducing podocyte oxidative stress (Fig. 8G). Glomerular TSP-1, TGF-β1 and phosphorylated Smad3 levels were examined by western blotting (Fig. 8C–F). TSP-1, TGF-β1 and phosphorylated Smad3 levels were upregulated in glomeruli of diabetic WT mice; however, this upregulation was inhibited by Sestrin2 (Fig. 8C–F). Additionally, the results of double immunofluorescence staining were consistent with the Western blot results (Fig. S10A, B, and S11A). These results indicate that Sestrin2 alleviates diabetes-induced podocyte injury by modulating TSP-1/TGF-β1/Smad3 signaling pathway.

## Discussion

DKD has become a serious health concern [22], and evidence indicates that podocyte injury is a crucial segment of DKD pathogenesis [23]. Studies have demonstrated that oxidative stress is strongly associated with podocyte damage [24–26]. Sestrin2 is recognized as a highly conserved gene that is sensitive to stress or DNA damage [27]. It has been reported that Sestrin2 overexpression may cause AMPK pathway activation, alleviate HG-induced eNOS dysfunction and inhibit HG-induced mitochondrial dysfunction and apoptosis in podocytes [17, 28]. In addition, the expression of Sestrin2 is downregulated in glomerular parietal epithelial cells and is associated with increased proteinuria in three models of nephrotic syndrome, including adriamycin nephropathy, crescentic glomerulonephritis and puromycin aminonucleoside nephropathy, suggesting that low Sestrin2 expression might be a marker of glomerular epithelial cell injury [29]. In our study, the expression of Sestrin2 in glomeruli and tubules was much lower in DKD patients than in normal control group. Besides, Sestrin2 expression in glomerular podocytes was decreased in diabetic mice and Sestrin2 levels decreased gradually with time in podocytes after HG stimulation, indicating that low Sestrin2 expression might be a sign of podocyte injury in DKD.

Herein, we used B6-TgN (CMV-Sestrin2) mice, which overexpress Sestrin2, for subsequent analysis. We found that Sestrin2 completely reversed renal function impairment and renal pathological alterations in mice with STZ-induced diabetes, indicating that diabetes-induced renal lesions are mitigated by Sestrin2. Podocytes, which are anchored to GBM, take an important part in preventing albumin from entering the glomerular filtrate, and podocyte detachment from GBM and loss of heparan sulfate are directly linked to the urinary albumin excretion rate in both T1DM and T2DM [30–32]. Since glomerular function impairment is a contributor to diabetic nephropathy and podocytes are vulnerable to filtration barriers, we examined the change in podocyte number in renal tissues of mice. The results showed that podocyte number reduction was significantly reversed in diabetic TgN mice, indicating that Sestrin2 may have a critical effect on inhibiting podocyte loss in DKD.

Glomerular podocytes can undergo phenotypic conversion through a process known as EMT, which is characterized by loss of podocyte-specific marker expression and acquisition of transitional characteristics and ultimately results in glomerular filtration impairment, proteinuria and glomerulosclerosis [33]. Podocyte loss or a decrease in podocyte density is related to the development of disease [30, 31, 34], and podocyte detachment from GBM, as well as podocyte apoptosis in response to injury, are thought to be two key mechanisms of podocyte loss in glomerular disease [35]. In our present study, we found that overexpression of Sestrin2 obviously alleviated HG-induced podocyte phenotypic alterations and apoptosis and that interfering with Sestrin2 expression exacerbated these changes. Podocyte phenotypic conversion and changes in the expression of apoptosis-related proteins were obvious in diabetic WT mice and attenuated in diabetic TgN mice, suggesting that Sestrin2 plays an important role in preventing podocyte phenotypic alterations and apoptosis in DKD.

Moreover, we performed a proteomic study to better elucidate the underlying mechanism of the protective effect of Sestrin2 on podocyte injury induced by diabetes. A total of 36 differentially expressed proteins that were enriched in 15 distinct signaling pathways were identified in mouse podocytes in NG group, HG group and HG + OE group through mass spectrometry plus pathway enrichment analysis. The results suggest that TSP-1 and TGF-β signaling pathway are involved in the protective effect of Sestrin2. TSP-1 was the first TSP family member to be discovered and is an ECM protein that can mediate a variety of biological processes, transmit a series of signals and orchestrate interactions between cells [36]. Furthermore, TSP-1 can be secreted and released by several types of renal cells, including mesangial cells, podocytes, renal tubular epithelial cells, renal interstitial fibroblasts and infiltrating macrophages [37]. It has been documented that TSP-1 silencing can delay the progression of glomerulonephritis [38]. In addition, TSP-1 expression is upregulated in rats with unilateral ureteral obstruction, and mice with TSP-1 deficiency exhibit less inflammation and better preservation of renal tissue than WT mice [39]. In our study, Sestrin2 notably downregulated the increased expression of TSP-1 induced by HG and interacted with TSP-1 in podocytes exposed to HG. The antioxidant NAC markedly decreased HG-induced TSP-1 expression, and Sestrin2 overexpression reduced HG-induced ROS generation in podocytes. In addition, application of TSP-1 inhibitor restrained HG-induced podocyte injury. Therefore, we hypothesize that Sestrin2 might directly modulate HG-induced increase in TSP-1 expression or via downregulation of ROS production in podocytes.

TGF-β1 is a key modulator of renal fibrosis and excessive activation of TGF-β1 might result in progressive renal injury [40]; also, high expression of TGF-β1 in diabetic mice speeds up the progression of DKD [41]. TGF-β1 can promote ROS production by inducing the expression of Nox4 and inhibiting the activity of antioxidants, playing dual roles in renal fibrosis [42]. In addition, TSP-1 is considered as a crucial activator of TGF-β, and this effect may be mediated by CD36 receptor, resulting in TGF-β signaling pathway activation [43]. Blocking the binding of TSP-1 with CD36 can reduce free fatty acid-induced podocyte apoptosis [44], and TGF-β1 can promote podocyte phenotypic conversion [45]. Smad3 is a fibrogenic factor that can directly bind to the promoter region of collagen and participate in the generation and degradation of ECM [46]. Smad3 knockout significantly alleviates renal fibrosis in models of obstructive nephropathy, DKD, and other diseases, and TGF-β1 can inhibit the activity of antioxidant enzymes to mediate the occurrence of Smad3-dependent renal fibrosis [42]. In the present study, Sestrin2 overexpression or TSP-1 blockade effectively inhibited HG-induced activation of TGF-β1/Smad3 pathway in podocytes, and Pirfenidone, a TGF-β/Smad inhibitor, mitigated HG-induced podocyte injury, indicating that Sestrin2 inhibits podocyte phenotypic alterations and podocyte apoptosis by modulating the activation of TSP-1/TGF-β1/Smad3 pathway in DKD.

Collectively, our study shows that Sestrin2 has a protective effect against podocyte injury in DKD. In vivo data proved that TgN-Sestrin2 mice with STZ-induced diabetes were protected from renal lesions, podocyte loss, podocyte phenotypic alterations, increased expression of apoptosis-associated proteins, oxidative stress, and TSP-1/TGF-β1/Smad3 activation. In vitro data demonstrated that Sestrin2 overexpression arrested podocyte EMT, apoptosis and oxidative stress induced by HG and that Sestrin2 ameliorated podocyte injury via modulating TSP-1/TGF-β1/Smad3 axis. Therefore, targeting Sestrin2 may be a promising approach for the treatment of DKD.

## Materials and Methods

### Reagents

d-glucose, mannitol and N-acetylcysteine (NAC) were purchased from Sigma (St. Louis, MO). RPMI 1640 culture medium and fetal bovine serum (FBS) were obtained from Gibco Company (Gaithersburg, MD). Penicillin, streptomycin and TRIzol reagent were bought from Invitrogen Life Technologies (Carlsbad, CA). LSKL and Pirfenidone were obtained from MedChemExpress (NJ, USA). Recombinant mouse γ-interferon was bought from PeproTech (NJ, USA). Sestrin2 pcDNA plasmid, Sestrin2 shRNA plasmid and control plasmid were obtained from GenePharma (Shanghai, China). FuGENE® HD Transfection Reagent and the reverse transcription system were bought from Promega Corporation (Madison, WI), and SYBR Premix Ex TaqTMII was from Takara (Shiga, Japan). Antibodies against Sestrin2 (10795-1-AP), desmin (16520-1-AP), E-cadherin (20874-1-AP), synaptopodin (67339-1-Ig), Bax (50599-2-Ig), Bcl-2 (26593-1-AP), Nox4 (14347-1-AP), CD36 (18836-1-AP), TGF-β1 (21898-1-AP), Smad3 (66516-1-Ig), Cadherin-16 (15107-1-AP) and β-actin (20536-1-AP) were purchased from Proteintech (Chicago, IL), while antibodies against α-SMA (ab124964), nephrin (ab216692), TSP-1 (ab267388) and Wilms’ Tumor Antigen-1 (WT-1) (ab89901) were obtained from Abcam (Cambridge, UK) and antibodies for Cleaved Caspase-3 (9664) and p-Smad3 (9520) were from Cell Signaling Technology (Boston, USA). PE Annexin V Apoptosis Detection Kit l was purchased from Becton, Dickinson, and Company (NJ, USA). Streptozotocin (STZ) was provided by Sigma-Aldrich® (St. Louis, MO). Besides, biochemical detection kits for 24-h urinary protein (Upro), serum creatinine (Scr), blood urea nitrogen (BUN), and triglyceride (TG) were purchased from Nanjing Jiancheng Bioengineering Institute (Nanjing, China). 8-Hydroxydeoxyguanosine (8-OHdG) ELISA Kit was from Elabscience Biotechnology (Wuhan, China) and Mouse SESN2 (Sestrin2) ELISA Kit was obtained from Fine Test (Wuhan, China).

### Cell culture and transfection

Conditionally immortalized mouse podocytes were bought from the Cell Culture Centre (PUMC, CAMS, Beijing, China), cultured as previously described [47]. In this cell line, a temperature-sensitive SV40 large T-cell antigen (tsA58 Tag) is controlled by a γ-interferon-inducible H-2K^b^ promoter. In order to induce proliferation, cells were grown in RPMI 1640 culture medium at 33 °C, supplemented with 10% FBS, 100 U/ml penicillin and 100 mg/ml streptomycin, to which recombinant mouse γ-interferon 10 U/ml was added (growth permissive conditions). Then, podocytes were cultured at 37 °C and deprived of γ-interferon (growth restrictive conditions) for inducing quiescent and differentiated phenotype. When podocytes were 70–80% confluent, they were thermo-switched to 37 °C to differentiate for 14–20 days before experiments. Then, Stable transfections of podocytes with Sestrin2 pcDNA plasmid, Sestrin2 shRNA plasmid or control plasmid were conducted with FuGENE® HD Transfection Reagent according to the manufacturer’s instructions. The podocytes were then cultured in serum-free RPMI 1640 medium for 6 h, after that, the substrate was changed into the original medium. Afterward, the cells were stimulated with NG (5.6 mM), HG (30 mM), NG plus mannitol (24.4 mM) as an osmotic control, HG plus NAC (5 mM), HG plus LSKL (5 μM) or HG plus Pirfenidone (0.5 mg/ml) for 24 h.

### Animals and metabolic studies

Amplified from ConA-activated murine splenocytes, musculus Sestrin2 cDNA was cloned into eukaryotic expression vector pcDNA 3.1. The recombinant plasmid pcDNA 3.1-mSestrin2 was identified by sequencing and linearized by Pvu I. Afterwards, the linearized plasmid was purified in Sephadex G50 column and diluted with TE buffer to the final concentration of 5 ng/μl (1 mM Tris, 0.1 mM EDTA, pH = 7.4). Then, the solution was microinjected into the pronucleus of C57BL/6J fertilized eggs which were implanted in the oviducts of pseudo-pregnant recipient ICR mice. Subsequently, PCR was used to determine the genotype of DNA separated from the newborn mouse tail tissues and the congenic strain was developed by repeated back-crossing of the transgenic mice with C57BL/6J. Wild type (WT) littermates were utilized as controls. Mice were housed under standard conditions and provided chow and water *ad libitum* at the animal facilities of Hebei Medical University. All experimental animal procedures were reviewed and approved by Ethics Review Committee for Animal Experimentation of Hebei Medical University (No. IACUC-Hebmu-2021030). The mice were randomly divided into four groups: WT group (wild type mice, *n* = 7), WT + DM group (diabetic WT mice, *n* = 7), TgN group (B6-TgN (CMV-Sestrin2) mice, *n* = 7) and TgN+DM group (diabetic TgN mice, *n* = 7). Diabetes was induced at 8 weeks of age by intraperitoneal injection of STZ (50 mg/kg in fresh 0.1 M sodium citrate buffer, pH 4.5) for five consecutive days. Three days after the injection, the mice with blood glucose greater than 16.7 mmol/l were regarded as successful models. Control (nondiabetic) groups received citrate buffer. Blood glucose levels were monitored with a glucometer (Roche, Basel, Switzerland) every four weeks throughout the experimental period. Twelve or sixteen weeks after the onset of diabetes, the animals were placed in metabolic chambers for 24 hours to determine the volume of urine excretion. Thereby, urine was centrifuged at 1000 rpm for 1 minute to remove any debris and frozen at −80 °C for further analysis: Upro and 8-OHdG. Next, mice were sacrificed and blood was collected for subsequent examinations: Scr, BUN, TG. Meanwhile, renal tissues were collected for the experiments mentioned below. The equipment used to measure Upro, Scr, BUN, and TG was Biochrom Ultrospec 2100 pro (Biochrom Ltd., Cambridge, UK). Urinary assay for 8-OHdG was performed with the help of a microplate reader and Gen5 software (Biotek, Vermont, USA) according to the manufacturer’s guidelines.

### RNA extraction and quantitative RT-qPCR analysis

Total RNA from cells were isolated and prepared using TRIzol reagent. cDNA was synthesized using PrimeScriptTM RT reagent kit according to the manufacturer’s instructions. The specific primers for target genes, including α-SMA, E-cadherin, nephrin, desmin, synaptopodin, Bax, Bcl-2, Nox4, TSP-1, CD36, TGF-β1, Smad3, and 18s rRNA, were listed in Table 1. Real-time PCR was conducted in a 96-well optical reaction plate using SYBR Premix Ex TaqTMII. Real-time PCR reactions were conducted on Agilent Mx3000P QPCR Systems (Agilent, CA). Fold changes among gene expression were then calculated through 2^−ΔΔCT^ method.

**Table 1:** The sequences of PCR primer pairs used in this study.

Gene	Forward primers (5′–3′)	Reverse primers (5′–3′)
α-SMA	CGCCCTCGCCACCAGATCTG	TAGCCTTCATAGATGGGGAC
E-cadherin	GCCGGAGCCCTGCCACCCTG	CTTTCTGTAGGTGGAGTCCC
nephrin	GTTCCAAGCCAAAGGATGCC	GCATGTAATGCCAGGGCT
desmin	CCATTGCCCTGGGATGAACT	TACCCGATGCCCAGGTGATA
synaptopodin	GCCCTCCTTCTGCTTCAAGT	TCTTCCTCACTAAGCCCCGA
Bax	GCTTGCTTCAGGGTTTCATCCA	TGTCCACGGCGGCAATCATC
Bcl-2	GGCGGAGAACAGGGTACGATAAC	CGGGATGCGGCTGGATGGGG
TSP-1	ATGCCTGCGATGATGACGATGAC	ATACTGGGCTGGGTTGTAATGGAATG
CD36	GTCTATCTACGCTGTGTTCGGATCTG	TGTCTGGATTCTGGAGGGGTGATG
TGF-β1	ACCGCAACAACGCCATCTATGAG	GGCACTGCTTCCCGAATGTCTG
Smad3	AGACGCCAGTTCTACCTCCAGTG	GCCAGCAGGGAAGTTAGTGTTCTC
Nox4	CGAGCCAAAGGGGCCCTGAAG	AACAGCGTGCGTCTAACGGCA
18s	ACACGGACAGGATTGACAGA	GGACATCTAAGGGCATCACAG

### Western blot and co-immunoprecipitation analysis

Cells or tissues were collected with RIPA buffer, separated by SDS-PAGE, transferred to PVDF membrane (Millipore, Billerica, MA), and then incubated with primary antibodies (α-SMA, E-cadherin, nephrin, desmin, synaptopodin, Bax, Bcl-2, Cleaved Caspase-3, Nox4, TSP-1, TGF-β1, CD36, p-Smad3, Smad3, Sestrin2 and β-actin) in 5% milk overnight at 4 °C. The membranes were incubated with goat anti-rabbit or mouse IgG horseradish peroxidase conjugate, followed by scanning via Odyssey Fc System (LI-COR, USA). The intensity of bands was analyzed by Image J software (National Institutes of Health). As for Co-IP analysis, podocytes were lysed using lysis buffer (50 mM Tris/HCl, 1 mM EDTA, 150 mM NaCl, 0.25% sodium deoxycholate, 1% NP-40, 2 mg/mL aprotinin and 1 mM PMSF, pH 7.4) on ice for 30 min and centrifuged at 12,000 rpm for 20 min. Sestrin2 antibody (2 μg) was incubated with 30 μL Protein A&G magnetic beads (MCE, New Jersey, USA) with constant rotation for 2 h at room temperature. Next, sample lysates together with the magnetic beads were incubated overnight at 4 °C. Afterwards, the immunoprecipitated proteins were eluted and examined by Western blot using TSP-1 antibody, and the protein bands were detected via ECL detection system.

### Immunofluorescence staining, double immunofluorescence staining and confocal microscopy

Briefly, we fixed podocytes in six-well chamber slides with 4% paraformaldehyde for 20 min, followed by punching cell membrane of podocytes with Triton X-100. Next, cells were incubated with specific primary antibodies against α-SMA, E-cadherin, desmin, nephrin and synaptopodin overnight at 4 °C. Afterwards, the podocytes were exposed to IFKineTMgreen conjugated goat anti-rabbit IgG for 1 h at 37 °C. DAPI was utilized for cell nuclei (blue) staining at a concentration of 1.43 μM. At last, the cells were observed with a confocal microscope (Leica, Germany).

The frozen kidney tissues (at −80 °C) were embedded with compound’s O.C.T. from Sakura Finetek (Torrance, CA) (Optimal Cutting Temperature), and their sections (8 μm) were prepared. Subsequently, the sections were made natural air-dry and fixed in iced acetone for 20 min, followed by being blocked with normal goat serum (10%) for 30 min at 37 °C. Additionally, renal sections were incubated with primary antibodies against α-SMA, E-cadherin, Bcl-2, Cleaved Caspase-3, Bax, Nox4, TSP-1, TGF-β1, p-Smad3, Sestrin2 and synaptopodin overnight at 4 °C. Finally, the kidney tissues were incubated with IFKineTMgreen conjugated goat anti-rabbit IgG and IFKineTMred conjugated goat anti-mouse IgG at 37 °C for 2 h, and nuclei were counterstained using DAPI. The images were obtained through a confocal microscope.

### Isolation of glomeruli

Glomeruli were isolated through a modified approach as described in previous studies [48, 49]. Renal cortex of mice was grinded into small pieces in pre-cooled tubes and digested with collagenase I for 30 min at 37 °C with gentle agitation. Next, glomeruli were segregated from tubules by successive sieving via 105-, 75-, and 40-μm cell strainers. The glomeruli-rich tissues on the 40-μm cell strainer were rinsed in a cell culture dish to make tubules adhere rapidly to the dish, while leaving glomeruli floating, namely, differential adhesion. Then the floated glomerular fraction was rinsed again with the 40-μm strainer, followed by an additional differential adhesion to achieve highly purified glomeruli. Isolated glomeruli purity was further determined by high expression of the podocyte marker nephrin and without detectable tubular marker Cadherin-16 (Fig. S11B).

### Intracellular ROS detection

Intracellular ROS formation was determined using the fluorescence probe 5-(and 6) chloromethyl-2′, 7′-dichlorodihydrofluorescein diacetate (CM-DCHF-DA, Invitrogen, Carlsbad, CA). Cultured in six-well plates under different conditions for 24 h, the podocytes were then rinsed, trypsinized and suspended in PBS. In addition, the cells were loaded with DCHF-DA (10 μM) and incubated at 37 °C for half an hour. The detection of intracellular ROS formation was conducted by the aid of a flow cytometer (BD Immunocytometry Systems, Franklin Lakes, NJ).

### Tissue histology and immunohistochemistry

After fixed in 4% paraformaldehyde overnight, kidney tissues were embedded in paraffin and 3-μm sections were prepared. Renal sections were then deparaffinized with xylene and rehydrated by graded ethanol. Next, the internal peroxidase was deactivated using 3% hydrogen peroxide in methanol for half an hour and antigen retrieval was performed via autoclaving for 15 min at 121 °C in citric acid monohydrate solution (pH 6.0). Subsequently, the sections were blocked with 10% normal goat serum for 30 min at room temperature and incubated with primary antibodies against nephrin, synaptopodin, Sestrin2 and WT-1 at 4 °C overnight. Afterwards, renal sections were washed by PBS and incubated with biotinylated secondary antibody and horseradish peroxidase-conjugated streptavidin. Labeling was visualized through 3, 3-diaminobenzidine and the sections were counterstained with hematoxylin. Finally, the slides were photoed with the aid of an inverted microscope (Olympus BX63). Quantitative analysis was carried out on 10 glomeruli per kidney section via Image Pro Plus 6.0. The number of WT-1–positive cells per glomerulus were counted as previously described [50]. Periodic-acid-schiff (PAS) staining and Masson staining were performed using staining kits following manufacturer’s instructions (Solarbio, Beijing, China).

### TUNEL assay

Podocytes were grown and stimulated on plates with six chambers under different conditions for 24 h, and the number of apoptotic cells was estimated by TUNEL BrightGreen Apoptosis Detection Kit (Vazyme, China) according to its instructions. For quantifying TUNEL-positive signals, at least 300 cells were counted per well. Moreover, the percentage of positively labeled podocytes was calculated with the help of Image Pro Plus 6.0 software.

### Human renal biopsy samples

Human renal biopsy tissues were harvested in the Second Hospital of Hebei Medical University. This study was ratified by Hebei Medical University ethical committees. Informed consents were obtained from patients according to approved guidelines. In total, there were 14 renal biopsies from individual patients in our study: 11 samples were from individuals suffering from type 2 diabetes with DKD confirmed by biopsy and the rest samples were from normal control patients. The normal control kidney tissues were received from the intact normal pole of nephrectomy samples of individuals who suffered circumscribed solitary renal tumors on the opposite pole but without clinical or histologic renal disease. The renal biopsies in DKD group were conducted to eliminate the possibility of coexistence of other kidney diseases.

### Transmission electron microscopy

Renal tissues were collected and fixed in 2.5% glutaraldehyde at 4 °C. Renal sections were rinsed 15 min for 3 times with 0.1 mol/L PBS and post-fixed in 1% osmium tetroxide at room temperature for 2 h. Then, specimens were dehydrated with a series of graded ethyl alcohol solutions. After exchange through acetone, the sections were embedded in Pon 812 resin overnight at 37 °C and acetone was served as a transitional solvent. The ultra-thin sections were prepared and post-stained with 2% saturated uranyl acetate and lead citrate. To determine the GBM thickness, TEM images were analyzed using ImageJ software. Three glomeruli were randomly chosen from each mouse and five electron micrographs were taken in each glomerulus.

### Flow cytometric analysis

Cells with different stimulation were harvested, rinsed in iced PBS twice, and suspended again in 1 × Binding Buffer (1 × 10^6^ cells/mL final concentration). Then, 100 μL (1 × 10^5^ cells) of the solution was transferred to a culture tube (5 mL capacity), and Annexin V and 7-AAD (both 5 μL) were added to the solution. The solution was vortexed gently and stood still at room temperature for 15 min in absence of light. Finally, 1 × Binding Buffer (400 μL) was added into each tube and the stained cells were analyzed by FACS flow cytometer using FACSAria’s FCM analysis Multicycle AV software from BD Biosciences (Franklin Lakes, NJ).

### Measurement of Sestrin2 level in mouse serum

Enzyme-Linked Immune-Sorbent Assay (ELISA) kit was utilized to determine endogenous Sestrin2 levels in serum, according to the manufacturer’s instructions (Fine Test, China). Tetramethyl benzidine (TMB) substrate solution was employed to visualize HRP enzymatic reaction. Thereby, the intensity of yellow color was directly proportional to the concentration of Sestrin2 in the samples. The optical density (OD) was measured at 450 nm and Sestrin2 levels were calculated via standard curve.

### Statistical analysis

The results from at least three independent experiments were expressed as mean ± standard deviation (SD). Statistical analysis was performed by one-way ANOVA using Graphpad Prism 5.01. Comparison between two sets of samples was analyzed by *t* test. Statistical significance was defined as *P* < 0.05.

### Reporting summary

Further information on research design is available in the Nature Research Reporting Summary linked to this article.

## Supplementary information

Figure S1

Figure S2

Figure S3

Figure S4

Figure S5

Figure S6

Figure S7

Figure S8

Figure S9

Figure S10

Figure S11

Supplementary figure legends

Original bands

Reporting Summary

## Data Availability

The datasets used and/or analyzed during the current study are available from the corresponding author on reasonable request.
