# Supplementary figures and images for: Sestrin2 remedies podocyte injury via orchestrating TSP-1/TGF-β1/Smad3 axis in diabetic kidney disease
Source: Cell Death Dis. 2022 Jul 30;13(7):663. doi: 10.1038/s41419-022-05120-0 (PMC9338940; doi:10.1038/s41419-022-05120-0)

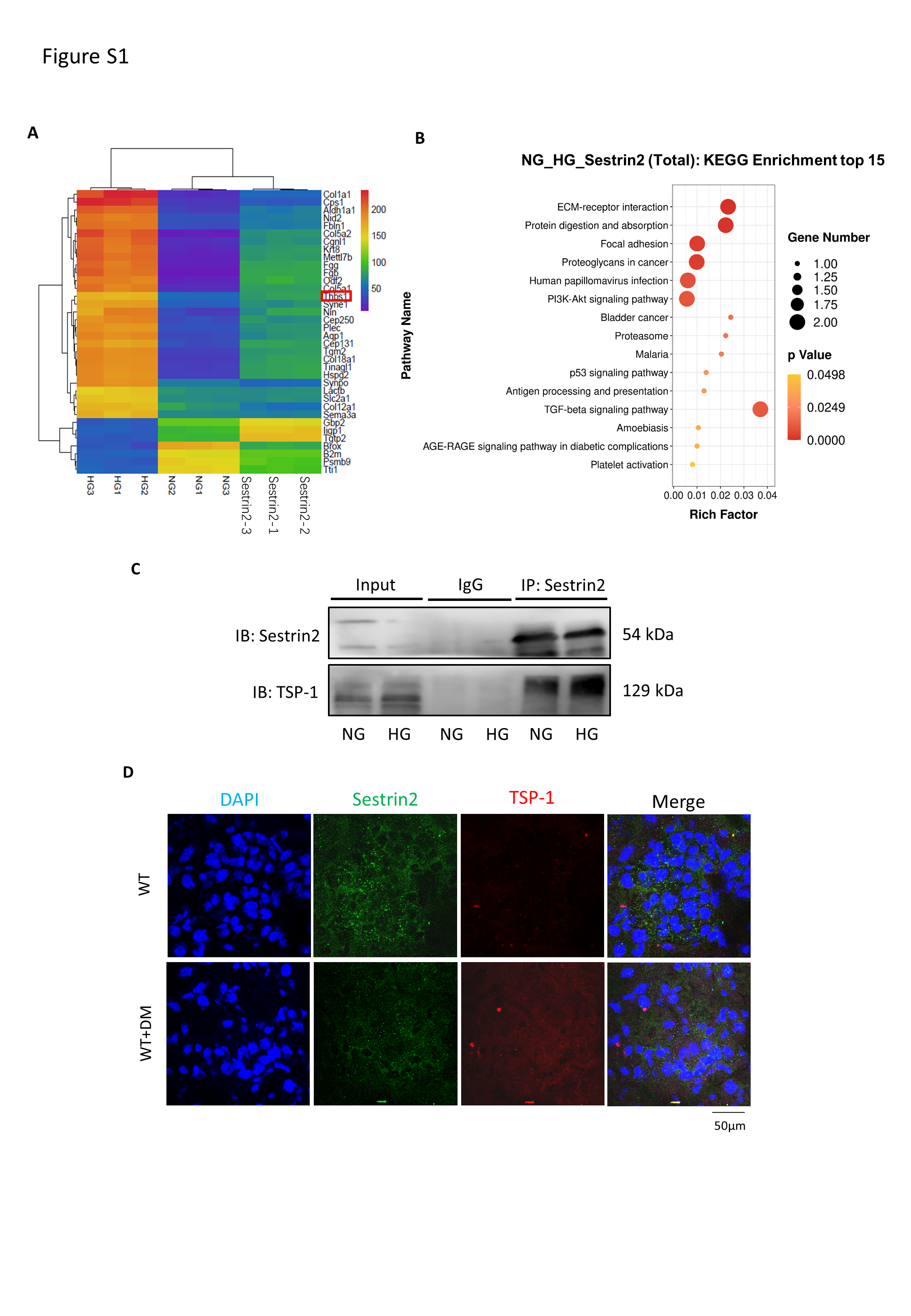

Supplement: Supplementary file 1 — Figure S1 [file 41419_2022_5120_MOESM1_ESM.tif]

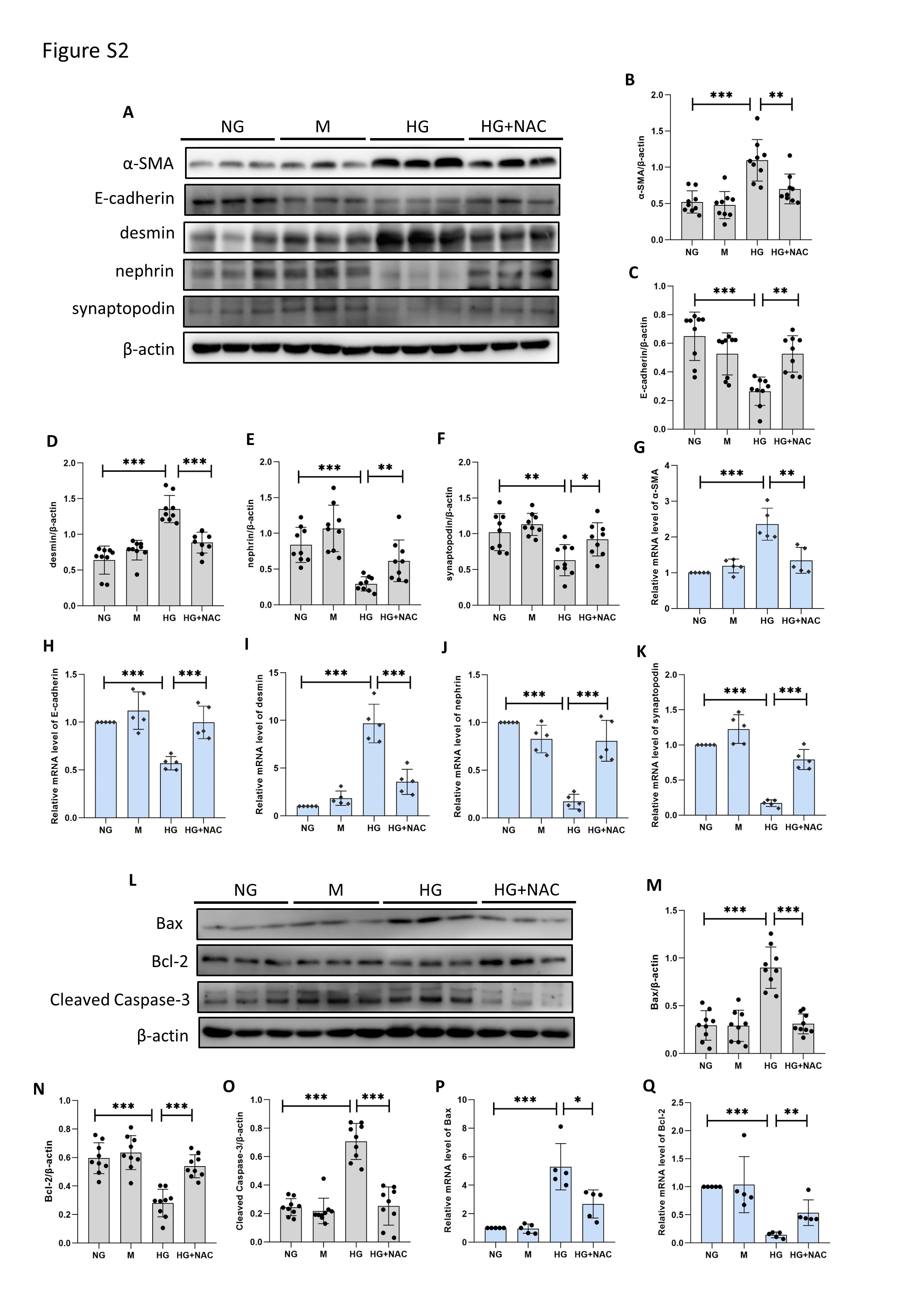

Supplement: Supplementary file 2 — Figure S2 [file 41419_2022_5120_MOESM2_ESM.tif]

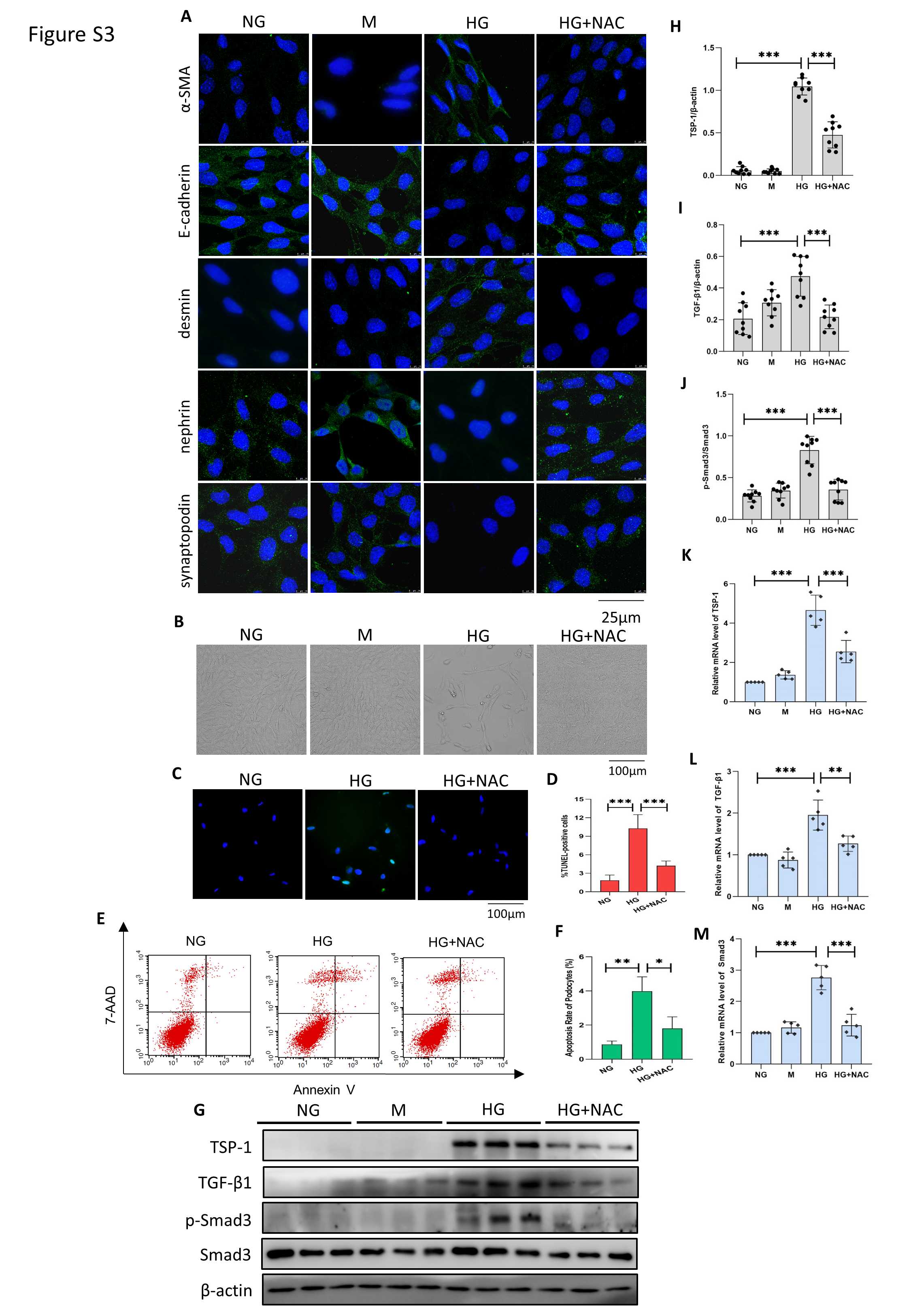

Supplement: Supplementary file 3 — Figure S3 [file 41419_2022_5120_MOESM3_ESM.tif]

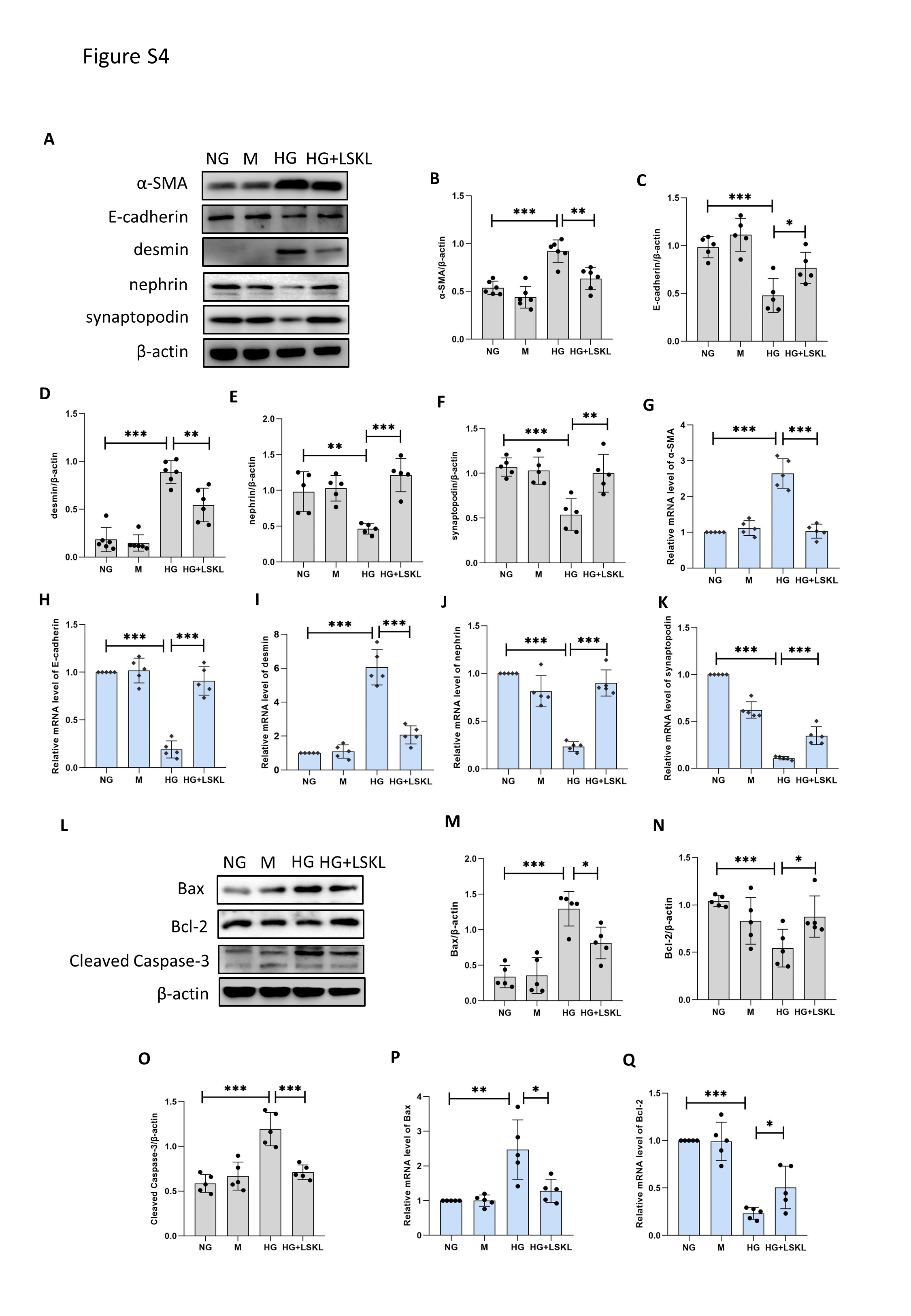

Supplement: Supplementary file 4 — Figure S4 [file 41419_2022_5120_MOESM4_ESM.tif]

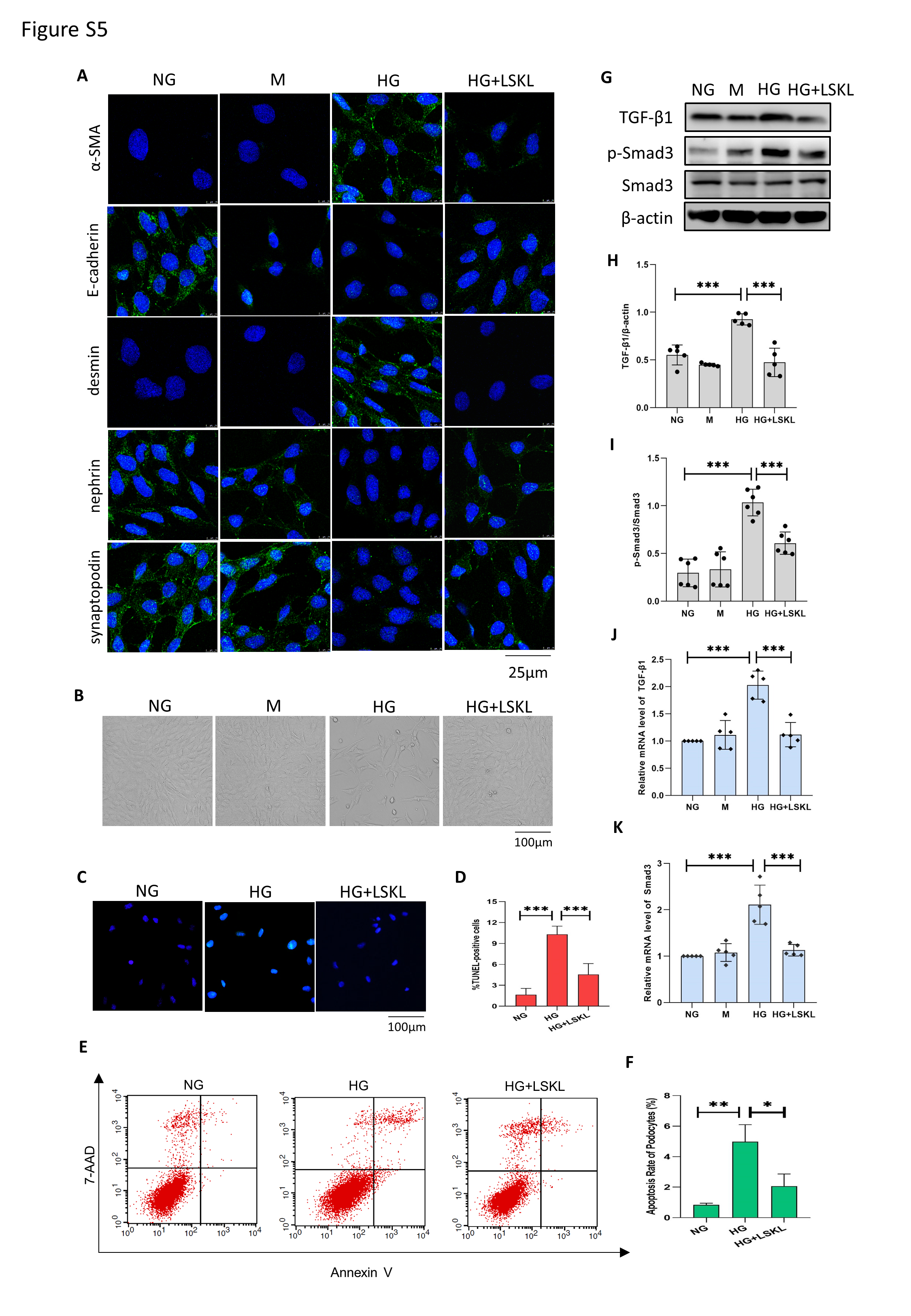

Supplement: Supplementary file 5 — Figure S5 [file 41419_2022_5120_MOESM5_ESM.tif]

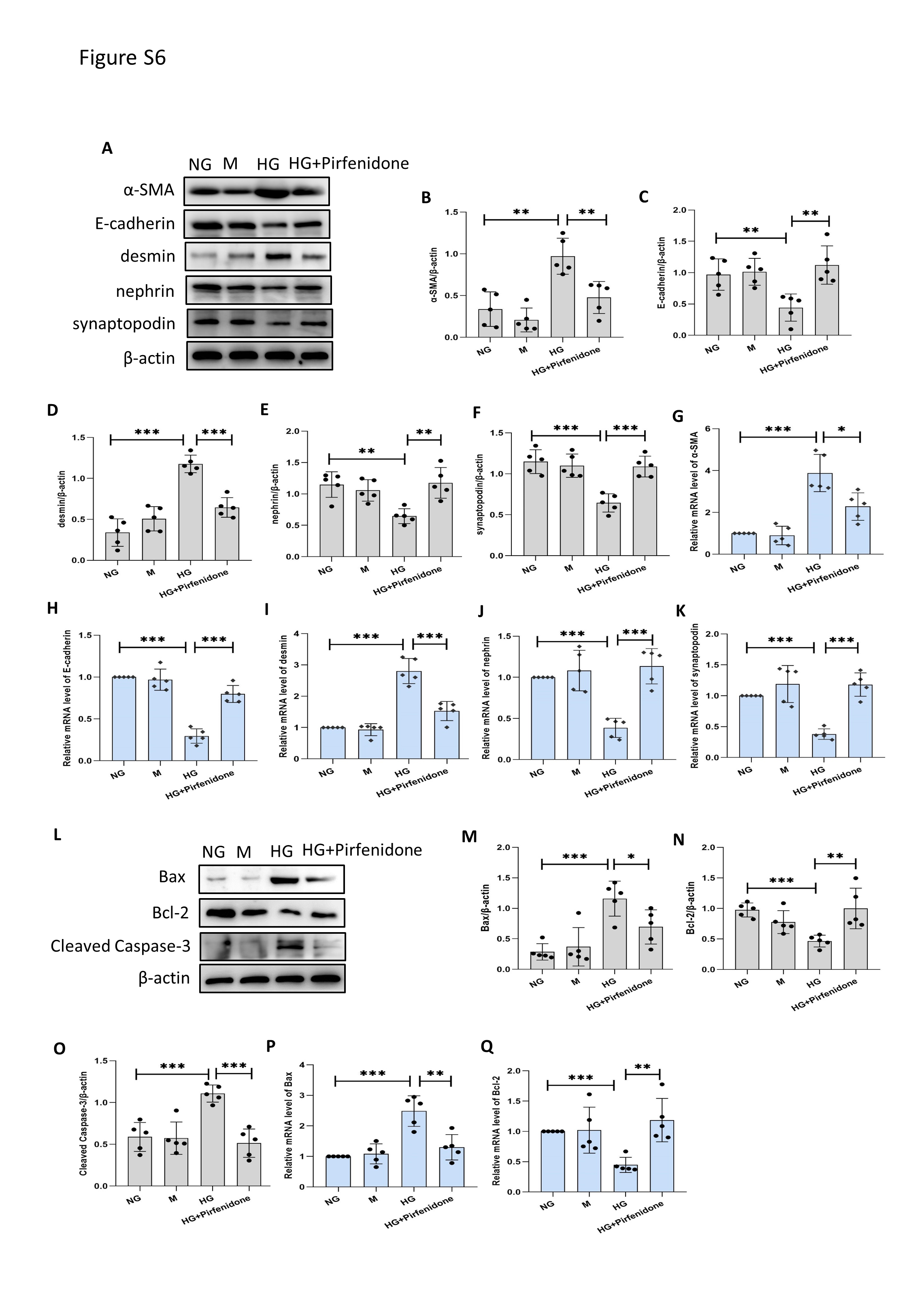

Supplement: Supplementary file 6 — Figure S6 [file 41419_2022_5120_MOESM6_ESM.tif]

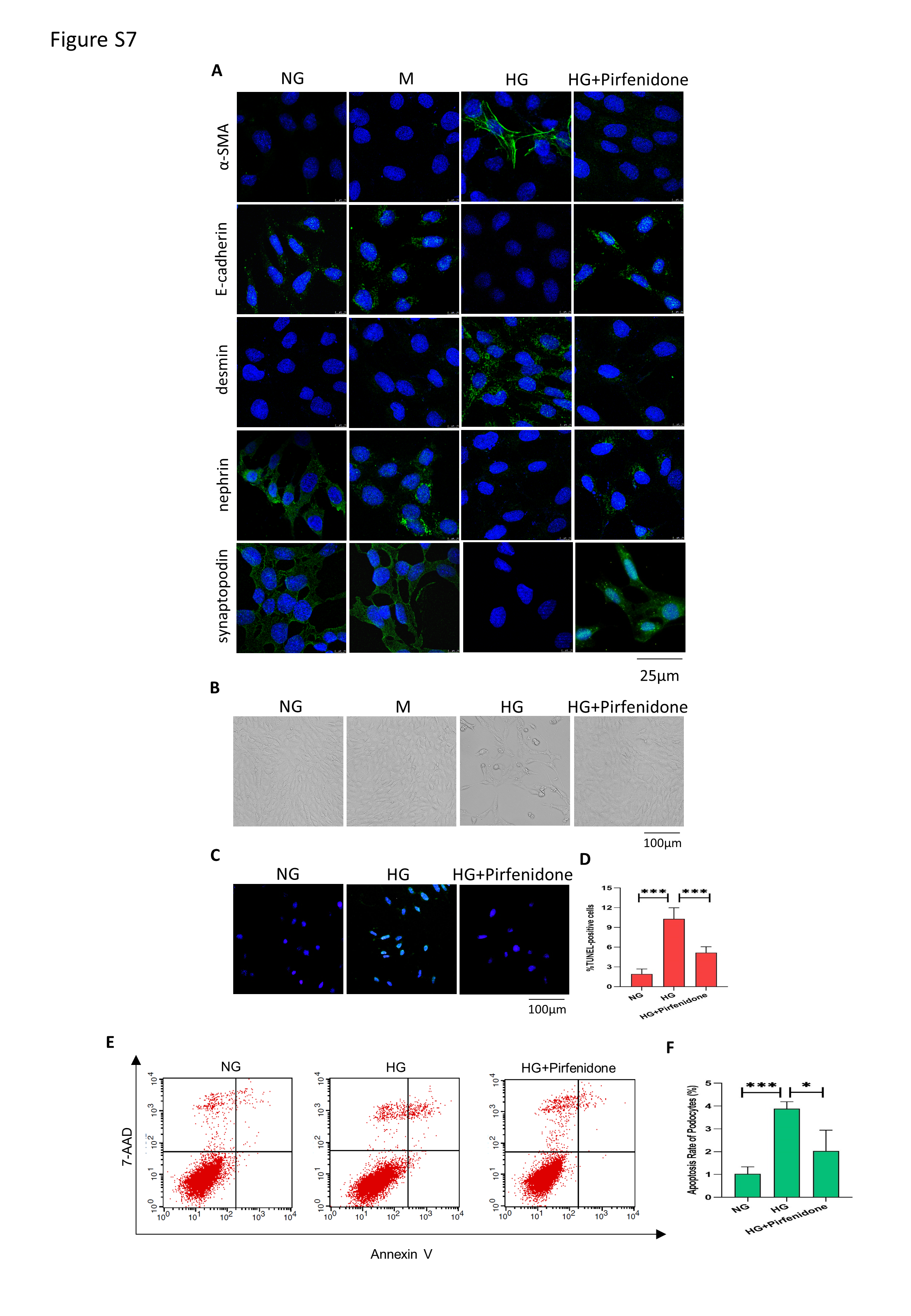

Supplement: Supplementary file 7 — Figure S7 [file 41419_2022_5120_MOESM7_ESM.tif]

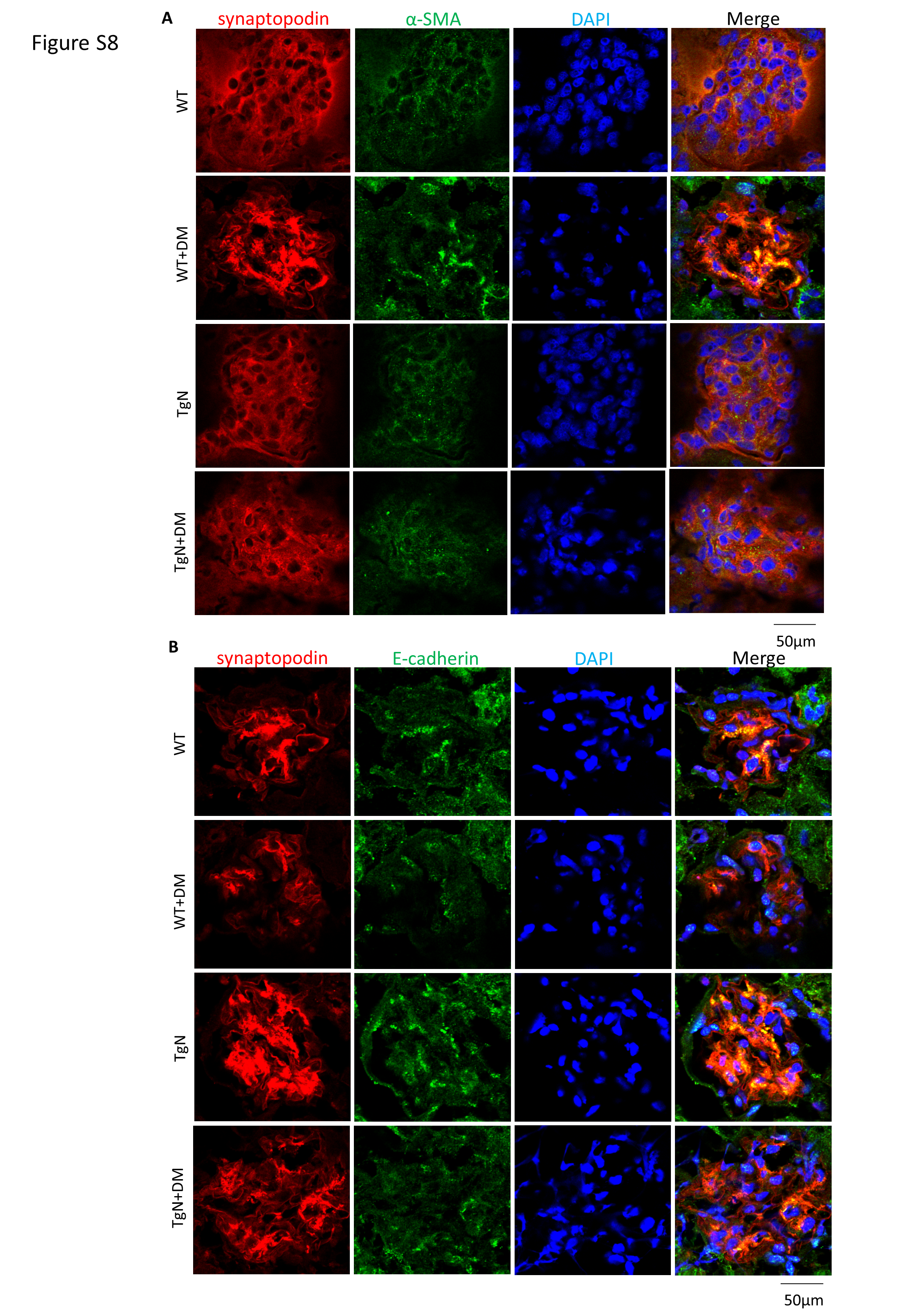

Supplement: Supplementary file 8 — Figure S8 [file 41419_2022_5120_MOESM8_ESM.tif]

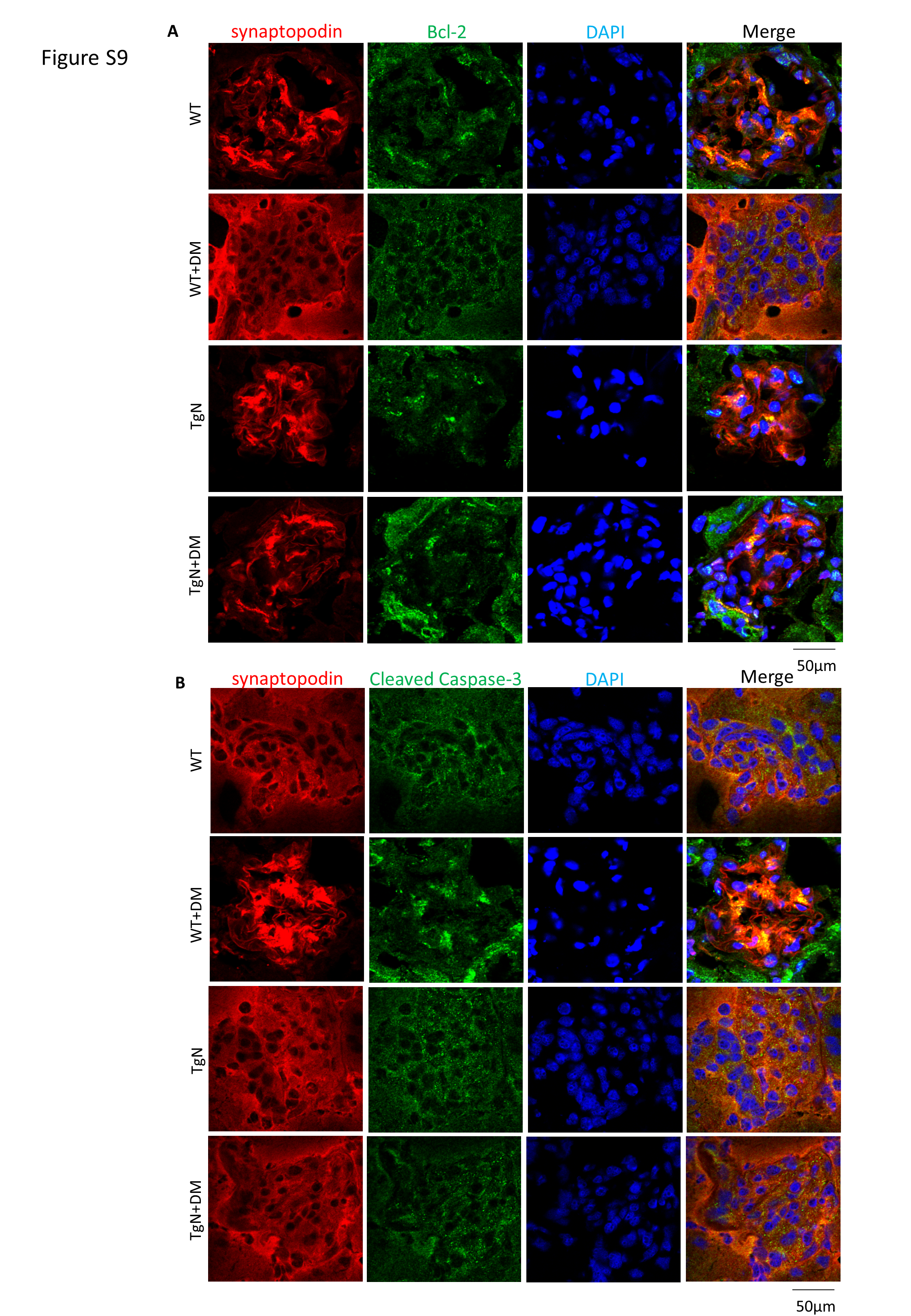

Supplement: Supplementary file 9 — Figure S9 [file 41419_2022_5120_MOESM9_ESM.tif]

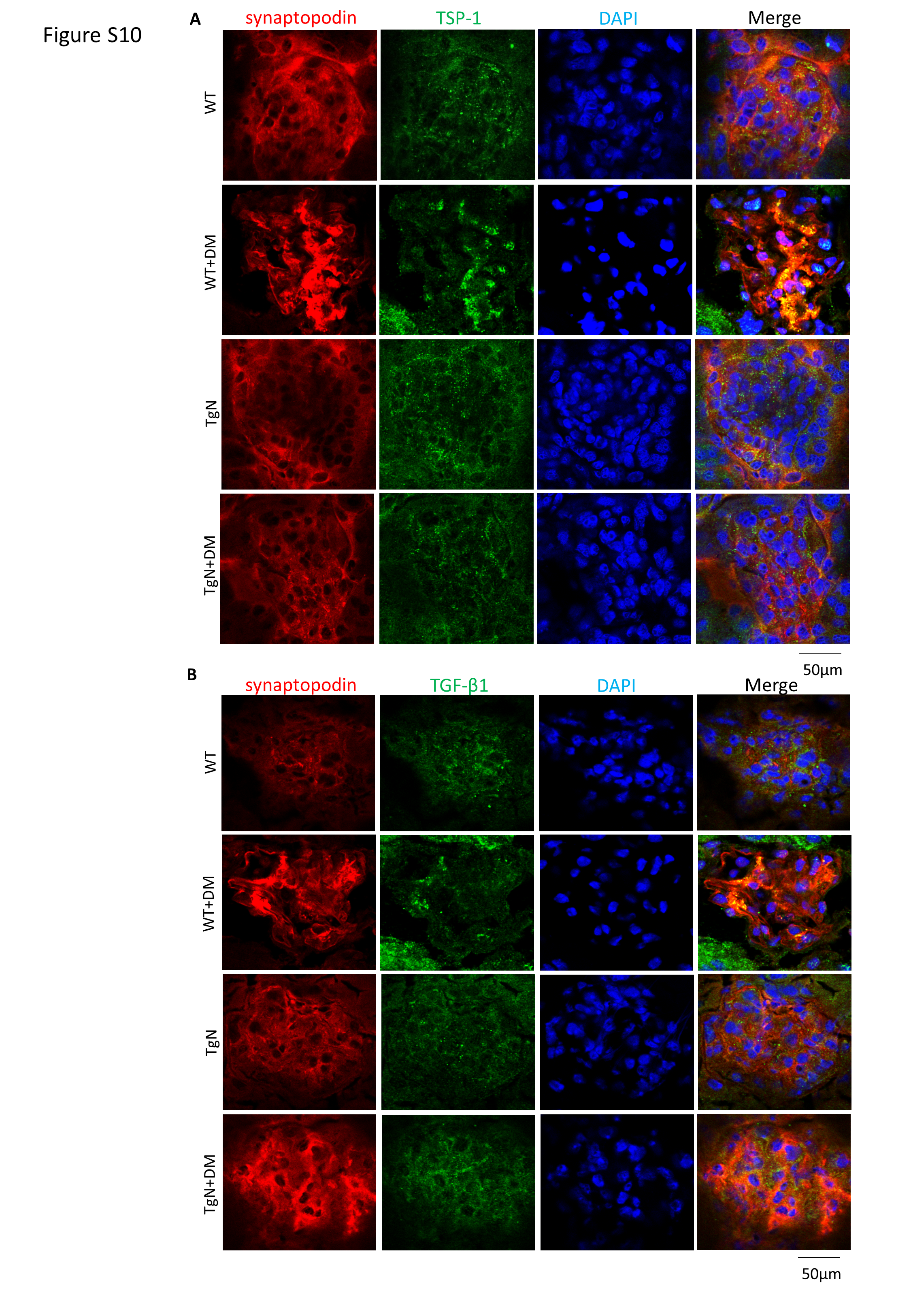

Supplement: Supplementary file 10 — Figure S10 [file 41419_2022_5120_MOESM10_ESM.tif]

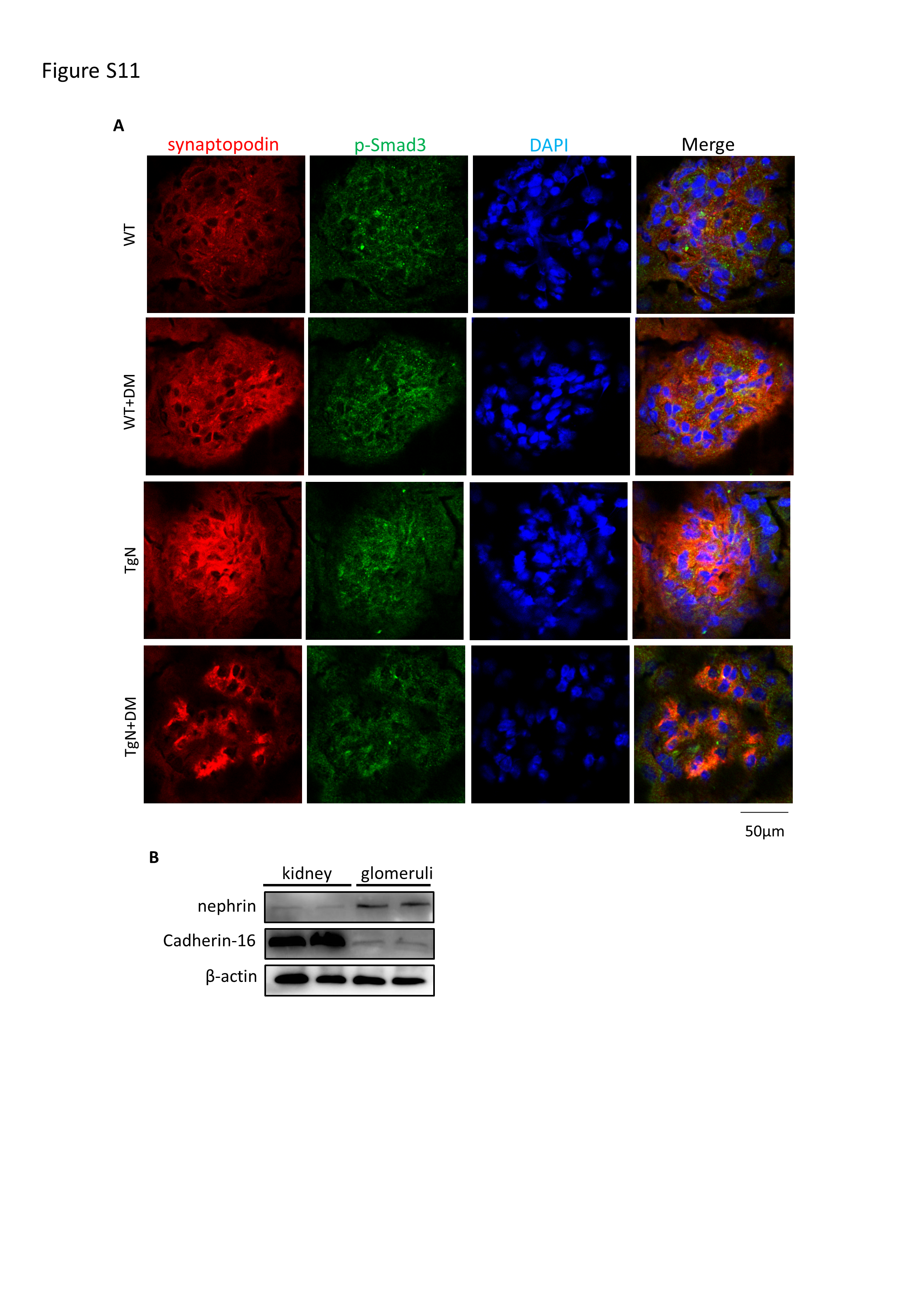

Supplement: Supplementary file 11 — Figure S11 [file 41419_2022_5120_MOESM11_ESM.tif]
