# Supplementary material for: Sestrin2 remedies podocyte injury via orchestrating TSP-1/TGF-β1/Smad3 axis in diabetic kidney disease
Source: Cell Death Dis. 2022 Jul 30;13(7):663. doi: 10.1038/s41419-022-05120-0 (PMC9338940; doi:10.1038/s41419-022-05120-0)
Supplement: Supplementary file 12 — Supplementary figure legends [file 41419_2022_5120_MOESM12_ESM.docx]

1. **Supplementary Figure Legends**

Figure S1. Sestrin2 tends to interact with TSP-1 in podocytes. (A) The heatmap for 36 distinct proteins screened from mouse podocytes of NG, HG and Sestrin2 group. (B) The bubble chart for top 15 potential relevant signaling pathways analyzed by KEGG enrichment. (C) Co-IP analysis demonstrated that Sestrin2 interacted with TSP-1 in podocytes. (D) Co-localization of Sestrin2 with TSP-1 in kidney tissues was detected by Immunofluorescence. DAPI is a nucleus dye.

NG: 5.6 mM D-glucose; HG: 30 mM D-glucose; Sestrin2: HG+Sestrin2 pcDNA. WT: wild type mice; WT+DM: diabetic WT mice. KEGG: Kyoto Encyclopedia of Genes and Genomes.

Figure S2. NAC shows a prohibitive effect on HG-induced podocyte EMT and apoptosis. (A-F) A representative Western blot and relevant quantification of α-SMA, E-cadherin, desmin, nephrin and synaptopodin in mouse podocytes (n=5). (G-K) The mRNA levels of α-SMA, E-cadherin, desmin, nephrin and synaptopodin were analyzed by RT-qPCR in mouse podocytes (n=5). (L-O) A representative Western blot and relevant quantification of Bax, Bcl-2 and Cleaved Caspase-3 in mouse podocytes (n=5). (P, Q) The mRNA levels of Bax and Bcl-2 were analyzed by RT-qPCR in mouse podocytes (n=5).

NG: 5.6 mM D-glucose; M: 5.6 mM D-glucose+24.4 mM mannitol; HG: 30 mM D-glucose; HG+NAC: HG+NAC (5 mM). Data are expressed as mean ± SD. *P<0.05, **P<0.01, ***P<0.001.

Figure S3. NAC shows an inhibitory effect on HG-induced podocyte EMT and apoptosis and activation of TSP-1/TGF-β1/Smad3 pathway. (A) Representative immunofluorescence images of α-SMA, E-cadherin, desmin, nephrin and synaptopodin in mouse podocytes, DAPI is a dye for nucleus. (B) Morphological changes of podocytes cultured under different conditions were analyzed by the inverted microscope. (C, D) TUNEL staining results for podocytes under different conditions (n=5). (E, F) The apoptosis rate of podocytes was detected by flow cytometry (n=3). (G-J) The protein levels of TSP-1, TGF-β1 and phosphorylation level of Smad3 were examined by Western blot in podocytes (n=5). (K-M) RT-qPCR was used to detect the mRNA levels of TSP-1, TGF-β1 and Smad3 in podocytes (n=5).

NG: 5.6 mM D-glucose; M: 5.6 mM D-glucose+24.4 mM mannitol; HG: 30 mM D-glucose; HG+NAC: HG+NAC (5 mM). Data are expressed as mean ± SD. *P<0.05, **P<0.01, ***P<0.001.

Figure S4. TSP-1 suppression inhibits podocyte EMT and apoptosis aroused by HG. (A-F) A representative Western blot and relevant quantification of α-SMA, E-cadherin, desmin, nephrin and synaptopodin in mouse podocytes (n=5). (G-K) The mRNA levels of α-SMA, E-cadherin, desmin, nephrin and synaptopodin were analyzed by RT-qPCR in mouse podocytes (n=5). (L-O) A representative Western blot and relevant quantification of Bax, Bcl-2 and Cleaved Caspase-3 in mouse podocytes (n=5). (P, Q) The mRNA levels of Bax and Bcl-2 were analyzed by RT-qPCR in mouse podocytes (n=5).

NG: 5.6 mM D-glucose; M: 5.6 mM D-glucose+24.4 mM mannitol; HG: 30 mM D-glucose; HG+LSKL: HG+LSKL (5 μM). Data are expressed as mean ± SD. *P<0.05, **P<0.01, ***P<0.001.

Figure S5. TSP-1 inhibition restrains podocyte EMT and apoptosis due to HG. (A) Representative immunofluorescence images of α-SMA, E-cadherin, desmin, nephrin and synaptopodin in mouse podocytes, DAPI is a dye for nucleus. (B) Morphological changes of podocytes cultured under different conditions were analyzed by the inverted microscope. (C, D) TUNEL staining results for podocytes under different conditions (n=5). (E, F) The apoptosis rate of podocytes was detected by flow cytometry (n=3). (G-I) The protein levels of TGF-β1 and phosphorylation level of Smad3 were examined by Western blot in podocytes (n=5). (J, K) RT-qPCR was used to detect the mRNA levels of TGF-β1 and Smad3 in podocytes (n=5).

NG: 5.6 mM D-glucose; M: 5.6 mM D-glucose+24.4 mM mannitol; HG: 30 mM D-glucose; HG+LSKL: HG+LSKL (5 μM). Data are expressed as mean ± SD. *P<0.05, **P<0.01, ***P<0.001.

Figure S6. TGF-β inhibition alleviates podocyte EMT and apoptosis induced by HG. (A-F) Representative Western blot and relevant quantification of α-SMA, E-cadherin, desmin, nephrin and synaptopodin in mouse podocytes (n=5). (G-K) The mRNA levels of α-SMA, E-cadherin, desmin, nephrin and synaptopodin were analyzed by RT-qPCR in mouse podocytes (n=5). (L-O) A representative Western blot and relevant quantification of Bax, Bcl-2 and Cleaved Caspase-3 in mouse podocytes (n=5). (P, Q) The mRNA levels of Bax and Bcl-2 were analyzed by RT-qPCR in mouse podocytes (n=5).

NG: 5.6 mM D-glucose; M: 5.6 mM D-glucose+24.4 mM mannitol; HG: 30 mM D-glucose; HG+Pirfenidone: HG+Pirfenidone (0.5 mg/ml). Data are expressed as mean ± SD. *P<0.05, **P<0.01, ***P<0.001.

Figure S7. TGF-β inhibition mitigates HG-induced podocyte EMT and apoptosis. (A) Representative immunofluorescence images of α-SMA, E-cadherin, desmin, nephrin and synaptopodin in mouse podocytes, DAPI is a dye for nucleus. (B) Morphological changes of podocytes cultured under different conditions were analyzed by the inverted microscope. (C, D) TUNEL staining results for podocytes under different conditions (n=5). (E, F) The apoptosis rate of podocytes was detected by flow cytometry (n=3).

NG: 5.6 mM D-glucose; M: 5.6 mM D-glucose+24.4 mM mannitol; HG: 30 mM D-glucose; HG+Pirfenidone: HG+Pirfenidone (0.5 mg/ml). Data are expressed as mean ± SD. *P<0.05, ***P<0.001.

Figure S8. Sestrin2 reverses podocyte phenotypic alterations of diabetic mice. (A, B) Representative immunofluorescence images of α-SMA (green), E-cadherin (green) and synaptopodin (red) in glomeruli from four groups of mice, and DAPI is a dye for nucleus.

WT: wild type mice; WT+DM: diabetic WT mice; TgN: B6-TgN (CMV-Sestrin2) mice; TgN+DM: diabetic TgN mice.

Figure S9. Sestrin2 reverts increased expression of apoptosis-associated proteins in podocytes of diabetic mice. (A, B) Representative immunofluorescence images of Bcl-2 (green), Cleaved Caspase-3 (green) and synaptopodin (red) in glomeruli from four groups of mice, and DAPI is a dye for nucleus.

WT: wild type mice; WT+DM: diabetic WT mice; TgN: B6-TgN (CMV-Sestrin2) mice; TgN+DM: diabetic TgN mice.

Figure S10. Sestrin2 orchestrates activation of TSP-1/TGF-β1/Smad3 signaling pathway induced by diabetes. (A, B) Representative immunofluorescence images of TSP-1 (green), TGF-β1 (green) and synaptopodin (red) in glomeruli from four groups of mice, and DAPI is a dye for nucleus.

WT: wild type mice; WT+DM: diabetic WT mice; TgN: B6-TgN (CMV-Sestrin2) mice; TgN+DM: diabetic TgN mice.

Figure S11. Sestrin2 regulates diabetes-induced activation of TSP-1/TGF-β1/Smad3 signaling pathway. (A) Representative immunofluorescence images of p-Smad3 (green) and synaptopodin (red) in glomeruli from four groups of mice, and DAPI is a dye for nucleus. (B) Western blot detected nephrin and Cadhein-16 expression in the whole kidney tissue and purified glomeruli.

WT: wild type mice; WT+DM: diabetic WT mice; TgN: B6-TgN (CMV-Sestrin2) mice; TgN+DM: diabetic TgN mice.
