# Supplementary material for: Sestrin2 remedies podocyte injury via orchestrating TSP-1/TGF-β1/Smad3 axis in diabetic kidney disease
Source: Cell Death Dis. 2022 Jul 30;13(7):663. doi: 10.1038/s41419-022-05120-0 (PMC9338940; doi:10.1038/s41419-022-05120-0)

Figure 1

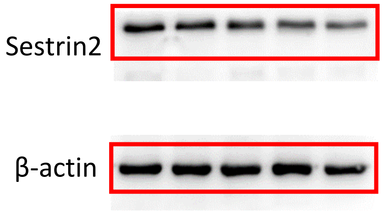

Figure 2

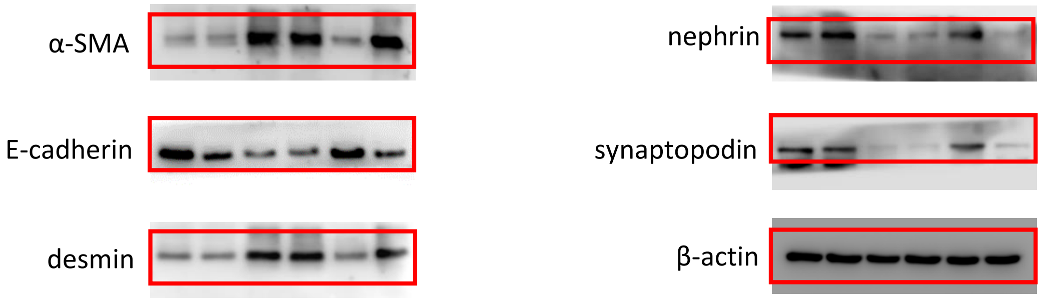

Figure 3

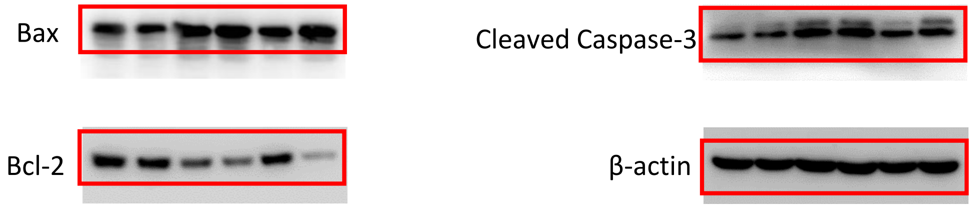

Figure 4A

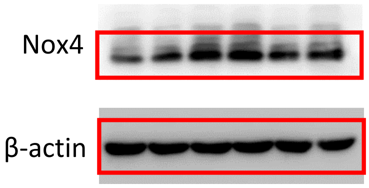

Figure 4D

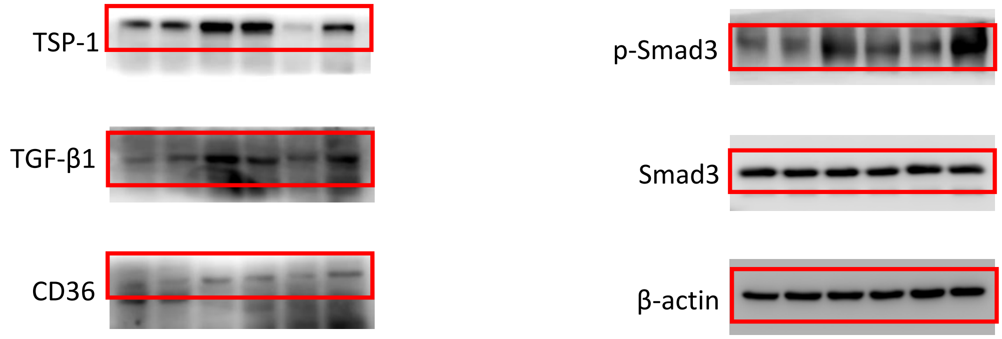

Figure 6

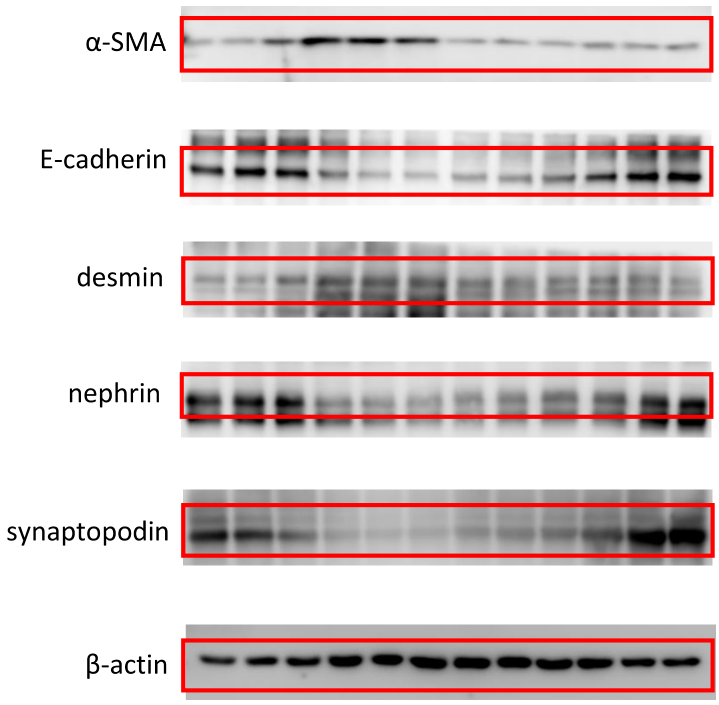

Figure 7

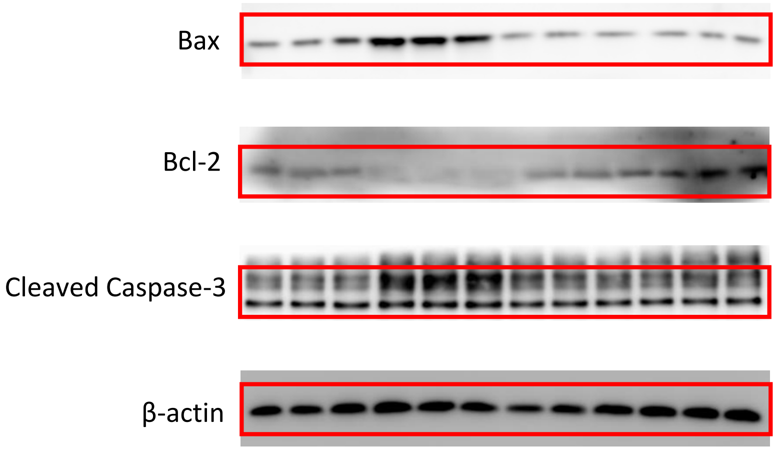

Figure 8A

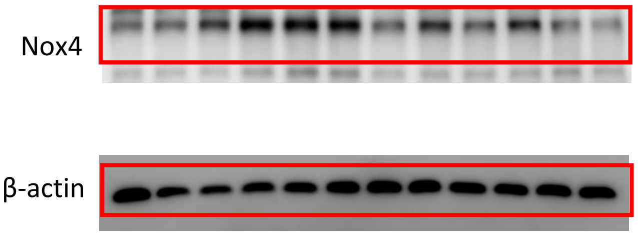

Figure 8C

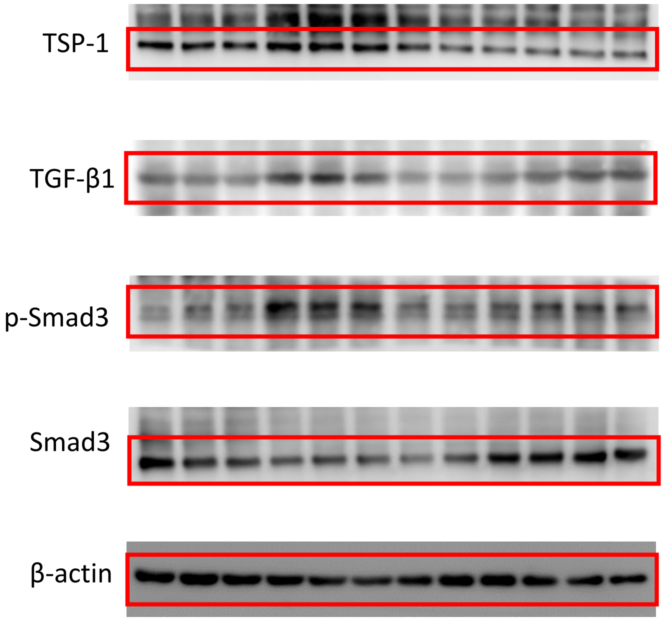

Figure S1

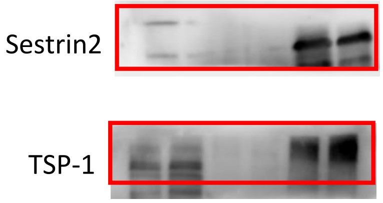

Figure S2A

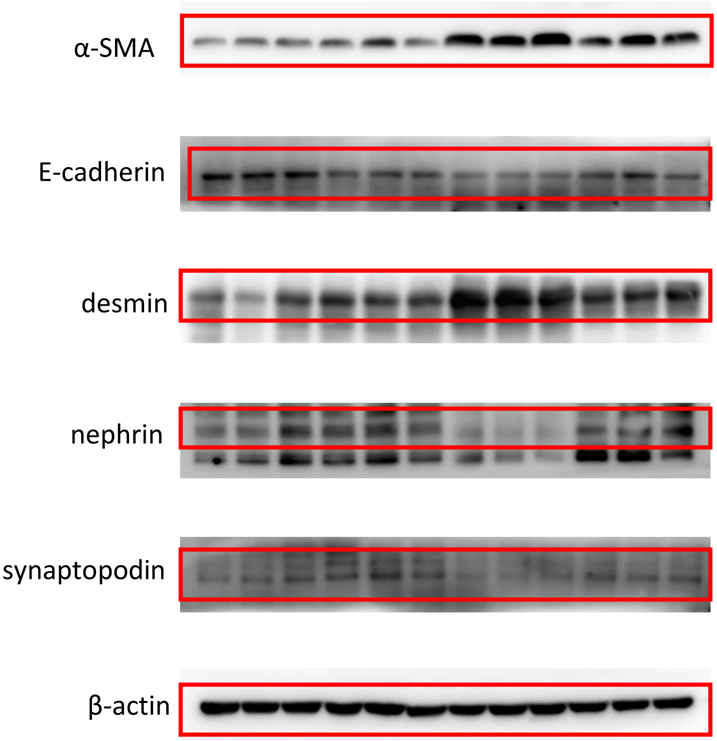

Figure S2L

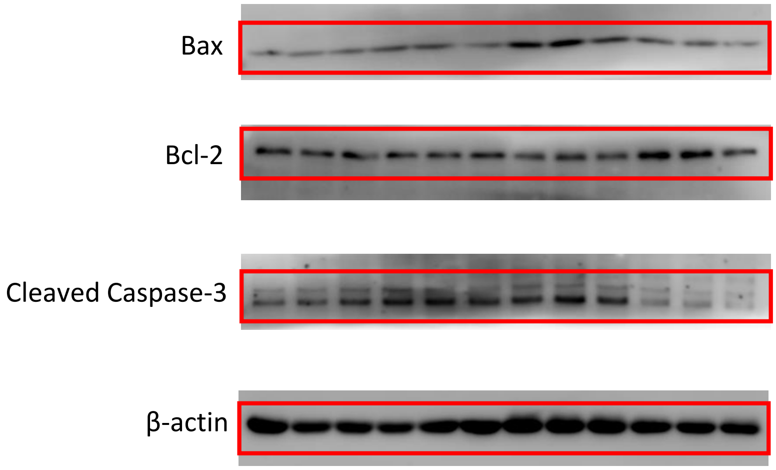

Figure S3

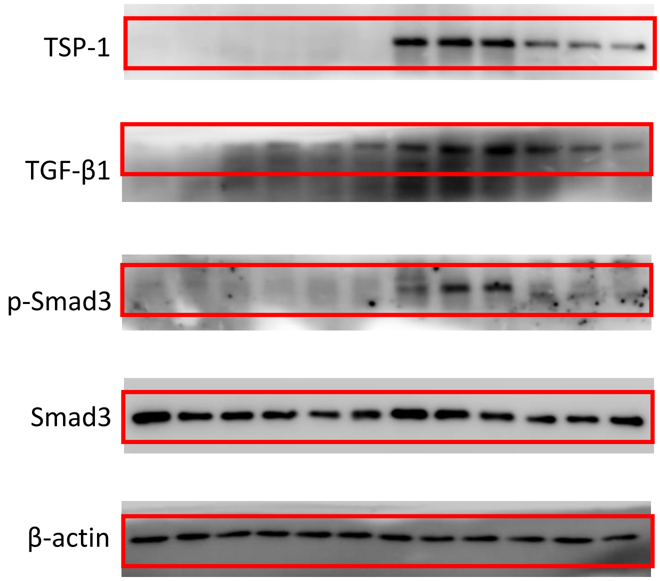

Figure S4A

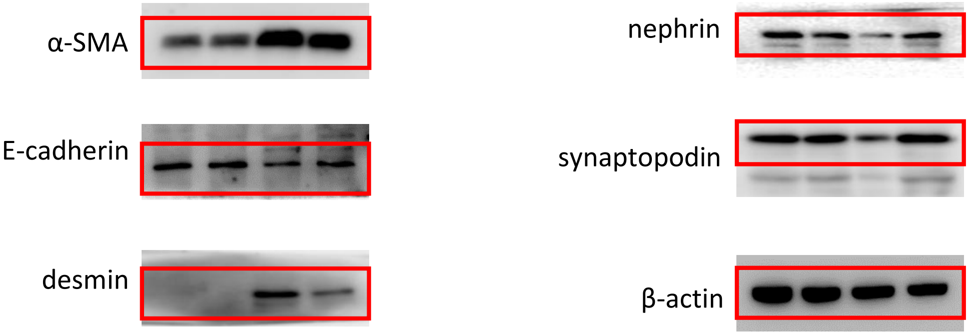

Figure S4L

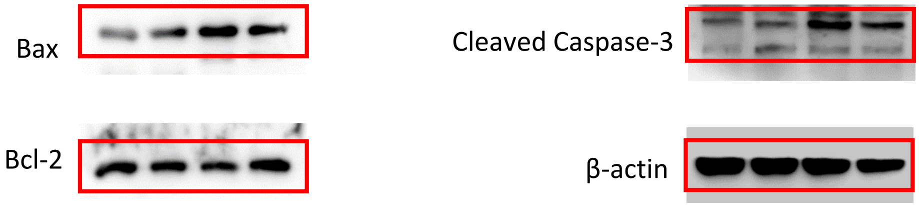

Figure S5

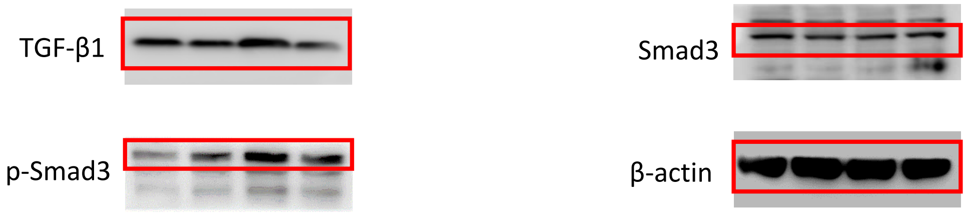

Figure S6A

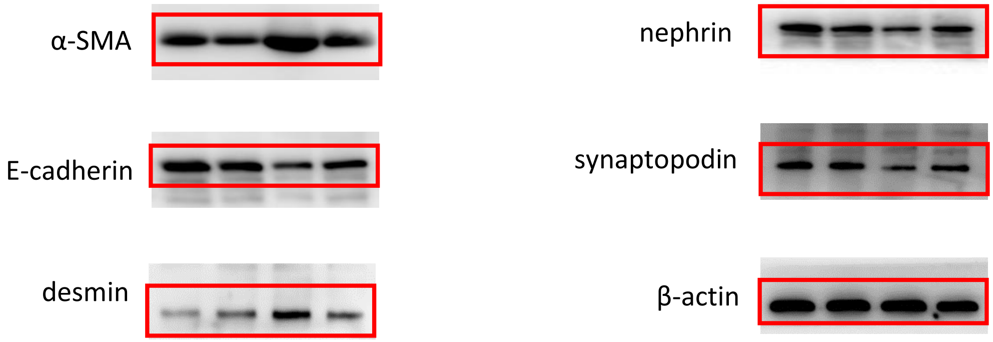

Figure S6L

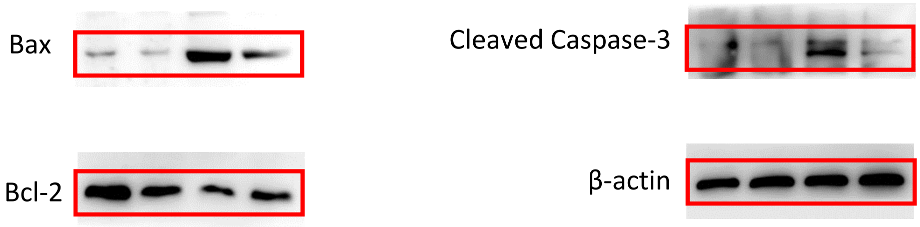

Figure S11B

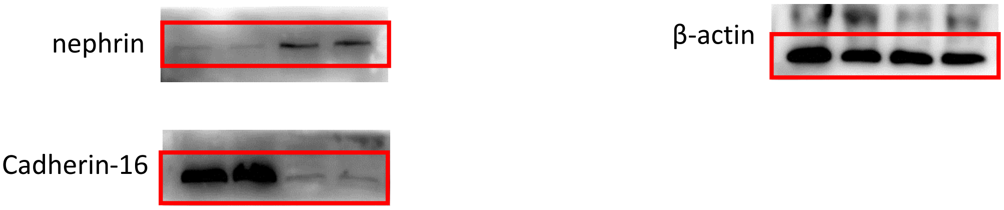

Supplement: Supplementary file 13 — Original bands [file 41419_2022_5120_MOESM13_ESM.pdf]
